# Characterizing the structural ensemble of γ-secretase using a multiscale molecular dynamics approach†
†Electronic supplementary information (ESI) available. See DOI: 10.1039/c7sc00980a



**DOI:** 10.1039/c7sc00980a

**Published:** 2017-06-05

**Authors:** Rodrigo Aguayo-Ortiz, Cecilia Chávez-García, John E. Straub, Laura Dominguez

**Affiliations:** a Departamento de Fisicoquímica , Facultad de Química , Universidad Nacional Autónoma de México , Mexico City , 04510 , Mexico . Email: lauradd@unam.mx; b Department of Chemistry , Boston University , Boston , Massachusetts 02215 , USA

## Abstract

γ-Secretase is an intramembrane-cleaving aspartyl protease that plays an essential role in the processing of a variety of integral membrane proteins. Its role in the ultimate cleavage step in the processing of amyloid precursor protein to form amyloid-β (Aβ) peptide makes it an important therapeutic target in Alzheimer's disease research. Significant recent advances have been made in structural studies of this critical membrane protein complex. However, details of the mechanism of activation of the enzyme complex remain unclear. Using a multiscale computational modeling approach, combining multiple coarse-grained microsecond dynamic trajectories with all-atom models, the structure and two conformational states of the γ-secretase complex were evaluated. The transition between enzymatic state 1 and state 2 is shown to critically depend on the protonation states of the key catalytic residues Asp257 and Asp385 in the active site domain. The active site formation, related to our γ-secretase state 2, is observed to involve a concerted movement of four transmembrane helices from the catalytic subunit, resulting in the required localization of the catalytic residues. Global analysis of the structural ensemble of the enzyme complex was used to identify collective fluctuations important to the mechanism of substrate recognition and demonstrate that the corresponding fluctuations observed were uncorrelated with structural changes associated with enzyme activation. Overall, this computational study provides essential insight into the role of structure and dynamics in the activation and function of γ-secretase.

## Abstract 2

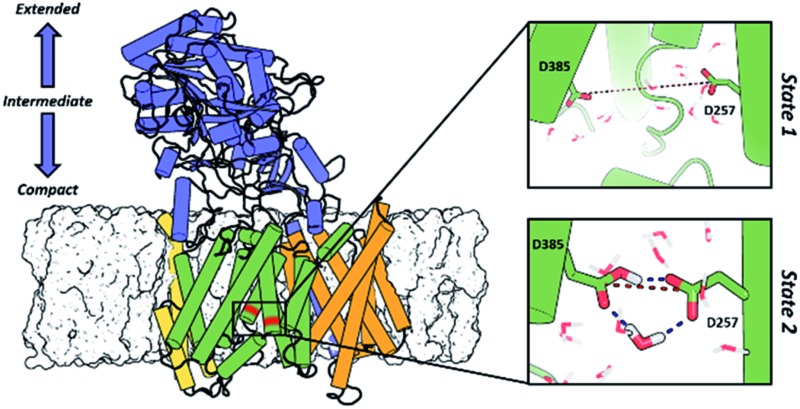
Employing a multiscale modeling approach, we characterized the structure and dynamics of the γ-secretase complex to elucidate its activation mechanism.

## Introduction

γ-Secretase is a membrane-embedded aspartyl protease that cleaves different integral membrane proteins within the lipid bilayer including amyloid precursor protein (APP), Notch, N-cadherin and ErbB4.^1^ APP is initially cleaved by β-secretase to release the APP C-terminal fragment (APP-C99), which is subsequently processed by γ-secretase to yield a variety of amyloid-β (Aβ) peptides of different lengths. The pattern of cleavage of APP-C99 results in a primary isoform, Aβ40 consisting of 40 amino acids, in addition to minor isoforms including Aβ38 and Aβ42.^2^ Aberrant accumulation of Aβ42 over Aβ40 has been associated with the formation of amyloid-β plaques in the brain of Alzheimer's disease (AD) patients.^2,3^ As such, developing an understanding of the mechanism of cleavage of APP-C99 by γ-secretase is an important goal for the field.

**Fig. 1:**
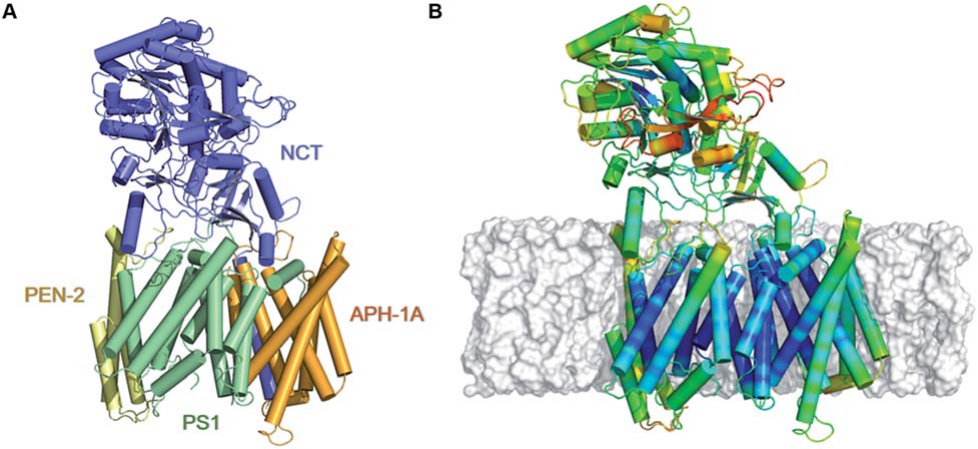
(A) Depiction of the all atom model of γ-secretase, derived from the 5FN2 PDB structure, colored by its subunits: **PS1**, **NCT**, **PEN-2**, and **APH-1A** in green, blue, yellow, and orange, respectively. (B) Fluctuation analysis of 5FN2-derived atomistic model of γ-secretase in POPC bilayer (gray) color-coded by the normalized per-residue root mean square fluctuation (RMSF) from more flexible (red) to less flexible (blue). The analysis was performed during the last 500 ns of the simulation.

The mature γ-secretase consists of four components (Fig. 1A): presenilin 1 (**PS1**), presenilin enhancer 2 (**PEN-2**), anterior pharynx-defective 1A (APH-1A) and nicastrin (NCT).^4–6^ **PS1** is the catalytic component of γ-secretase. It contains nine transmembrane helices (TMs) organized into a horseshoe-shaped structure with two catalytic Asp residues located at TM6 (Asp257) and TM7 (Asp385) within the convex side of the protein surface.^7–10^ Autoproteolysis of the intracellular loop connecting TM6 and TM7 leads to the formation of the **PS1** N-terminal (NTF, TMs 1–6) and C-terminal fragments (CTF, TMs 7–9) that interact with **PEN-2** and **APH-1A**, respectively.^9^ **PEN-2** consists of three TMs, of which TM1 and TM2 form a re-entrant loop extending halfway through the membrane from the intracellular side.^7,11^ The association of **PEN-2** with **PS1** has been related to the autocatalytic maturation of **PS1** and γ-secretase activity. **APH-1A** consists of seven TMs helices and a C-terminal juxtamembrane region.^12,13^ Several experimental studies suggest that this component is required for proper γ-secretase assembly.^14,15^ **NCT** is a transmembrane glycoprotein with a large N-terminal extracellular domain (ECD) and a single TM helix located in its C-terminal region.^16,17^ The globular ECD is comprised of a large and a small lobe composed of α-helices and β-strands.^12,18,19^ It has been proposed that Glu333 and Tyr337, located at **NCT**'s large lobe, interact with the substrate's extracellular domain and play a critical role in substrate recognition.^17,20^ These residues are buried in a hydrophilic pocket covered by a lid formed by the small lobe.^12^ It is thought that a rotation of **NCT**'s large lobe around a central pivot may cause lid opening, exposing the substrate-binding site.^17^ However, mutagenesis studies involving Glu333 contradict this theory, suggesting that Glu333 is involved in the maturation assembly of the complex rather than in substrate recognition.^21,22^

Novel high-resolution cryo-EM structures of human γ-secretase have been resolved.^7,12,19^ In the first proposed structure, Shi and coworkers^7^ reported the overall architecture of the complex at 4.5 Å resolution (PDB ID: ; 4UIS). Nevertheless, due to the limited resolution, partial assignment of the TMs side chains was only possible through sequence homology modeling of **PS1** using a presenilin homologue (PSH) (PDB ID: ; 4HYG).^8^ Subsequently, Bai, *et al.* ^12^ obtained an atomic-level γ-secretase structure with 3.4 Å resolution employing cryo-EM single particle analysis (PDB ID: ; 5A63). In this structure, the main-chain connectivity and side-chains were mostly displayed, facilitating identification of new interactions between the TMs, the presence of the juxtamembrane region of **APH-1A**, and a complete structure of the NCT ECD.

The structure of TM2 and the N-terminal region of TM6 in **PS1** are typically not resolved due to the high flexibility of these domains. Importantly, four new γ-secretase structures exhibiting varying conformational states in this critical region have been resolved (PDB IDs: ; 5FN2, ; 5FN3, ; 5FN4 and ; 5FN5).^19^ The first was obtained in complex with the dipeptidic inhibitor *N*-[*N*-(3,5-difluorophenacetyl)-*L*-alanyl]-*S*-phenylglycine *t*-butyl ester (DAPT); however, the structure of the inhibitor was not assigned. The second and third γ-secretase structures were solved in complex with peptide fragments. Interestingly, the authors suggest that the peptide fragment found in ; 5FN3, located between **PS1** TM2, TM3 and TM4, belongs to the N-terminal region of APP. Finally, the last structure did not appear to be complexed with any external agent, and as in the case of PDB ID: ; 5A63, it was not possible to resolve TM2.

A previous molecular dynamics (MD) study examined the dynamic properties and activation of a single human **PS1** subunit embedded in a variety of membrane lipid compositions, with an initial **PS1** conformation derived from homology modeling.^23^ However, in the absence of the **PEN-2**, **APH-1A**, and **NCT** subunits, the simulations exhibited large instabilities and flexibility in the TMs. More recently, Han and coworkers^24^ explored the initial substrate binding site of the transmembrane region of γ-secretase using a multiscale MD approach. In that work, the authors demonstrated the importance of using atomistic and coarse grained models to assess the behaviour of the TMs of forming the complex. Moreover, their results suggest that TM2/6/9 (including the PAL motif) constitute the initial APP-C99 binding site.

Despite these recent advances, many essential aspects of the structure and dynamics of the multicomponent γ-secretase complex remain undescribed. In this study, we employed a multiscale simulation approach that combines atomistic and coarse-grained models starting from a variety of cryo-EM structures to explore the dynamic structural ensemble of γ-secretase embedded in a POPC lipid bilayer. Conformational changes are analyzed using order parameters that characterize the essential dynamics of the enzyme complex, including the transition between active and inactive functional states. Our study provides critical insight into (1) the nature of large-scale conformational transitions in the γ-secretase complex, (2) the identification of two conformational states of **PS1**, constituting a mechanism for its activation, and (3) the characterization of NCT ECD mobility and its relationship to the observed **PS1** states.

## Methods

### Model preparation

Two cryo-EM structures of γ-secretase (PDB IDs: 5FN2 and ; 5FN3) resolved at 4.2 and 4.1 Å resolution, respectively, were used as initial 3D coordinates to perform the multiscale MD simulations of the γ-secretase complexes. The missing side-chains of the structures were completed using the WHAT IF web server.^25^ The spatial arrangement of the γ-secretase models in the POPC lipid bilayer was assigned with the Orientation of Proteins in Membranes (OPM) web server.^26^

### Molecular dynamics simulations

We used a multiscale computational approach (Table 1) to characterize the structure and dynamics of the γ-secretase enzyme complex embedded in a POPC bilayer, combining all-atom and CG representations, using CHARMM36 and MARTINI force field models, respectively, for the protein, membrane, and solvent environments. We simulated two distinct γ-secretase models, each in three different protonated states: two models with one of the two catalytic aspartic acid residues protonated (Asp257 and Asp385) and a third model with both catalytic residues unprotonated.

**Table 1:** Overview of all-atom and CG simulations employed in this study, color-coded according to the system model

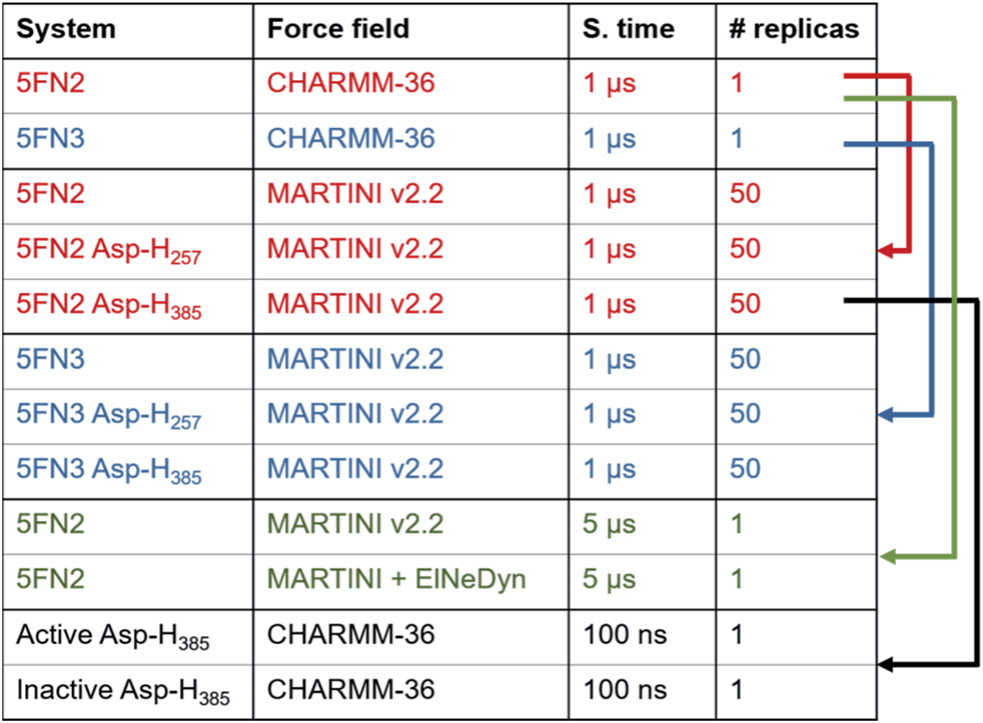

#### All-atom molecular dynamic simulations

Each protein was embedded in a palmitoyl oleoyl phosphocholine (POPC) membrane using the CHARMM-GUI membrane builder.^27,28^ The model of the ; 5FN2 derived system consisted of the reconstructed protein complex, 320 POPC lipids, 46 078 water particles, and 143 Cl^–^ and 148 Na^+^ ions; the ; 5FN3 derived system contained the reconstructed protein complex, 326 POPC lipids, 46 827 water particles, and 145 Cl^–^ and 150 Na^+^ ions. The systems were energy minimized and equilibrated with NVT and NPT dynamics. Equilibration of each simulation was followed by a 1 μs production run with a time step of 2 ps. The temperature was set to 310 K using the Nose–Hoover coupling thermostat algorithm and the pressure was set to 1.0 bar using the semi-isotropic Parrinello–Rahman barostat algorithm. The Lennard-Jones potential was truncated using a shift function between 0.9 and 1.2 nm. Full electrostatic interactions were calculated between 0 and 1.2 nm, after which the electrostatic interactions were calculated using the Particle Mesh Ewald (PME) approach. Neighbor lists were updated every 20 steps and bonds involving hydrogens were constrained using the Linear Constraint Solver (LINCS) algorithm.^29^ The simulations were performed using GROMACS 5.0.6 (ref. 30) with the CHARMM36 force field^31^ and the TIP3P water model. The apolar contribution to the energy assigned to interaction of PS with adjacent components was calculated with the g_mmpbsa.py^32^ script. Finally, the MD simulations were analyzed using the inbuilt GROMACS tools and the MdAnalysis libraries^33^ for python and the images were generated using Bendix,^34^ VMD v1.9.2 (ref. 35) and PyMOL v0.9 (ref. 36).

#### Coarse-grained (CG) model simulations

Three coarse-grained systems were built for each of the 5FN2 and ; 5FN3 derived γ-secretase models: two models with one of the two catalytic aspartic acid residues protonated (Asp257 and Asp385) and a third model with both catalytic residues in an unprotonated state. Each model protein complex was embedded in a POPC bilayer using the CHARMM-GUI Martini bilayer maker^37,38^ with the polarizable water model of the Martini v2.2 force field.^39^ The temperature was set to 310 K using V-rescale coupling and the pressure was set at 1.0 bar with a semi-isotropic Berendsen coupling. Trajectories for 50 replicas of the protonated and unprotonated states of the ; 5FN2 and ; 5FN3 derived models were performed for 1 μs of NPT dynamics for a total of 150 μs of dynamics for the membrane-embedded γ-secretase complex, time evolution of the CG trajectories suggested a broad sampling of the conformational space on each simulation (Fig. S4†).

Additionally, in order to evaluate the stability of our simulations and validate our methodology and results, two longer simulations of 5 μs were performed for the unprotonated γ-secretase complex using our CG Martini model and a CG Martini with an Elastic Network in Dynamics (CG-ElNeDyn).^40^ The systems were prepared following the same methodology described above and results are presented in the ESI.†

The most representative structures of the state 1 and state 2 **PS1** conformers from the ; 5FN2 derived CG simulations were selected using the GROMACS cluster tool with a root mean square deviation (RMSD) threshold of 2.0 Å. These structures were back-mapped from coarse-grained (MARTINI force field) models to all-atom (CHARMM36 force field) models using the script backward.py.^41^ The final systems were simulated for 100 ns of MD following the previously described all-atom simulation protocols.

Finally, three structures were selected from the 5FN2 derived CG simulations with protonated Asp385 to represent the compact, intermediate, and extended conformations of the γ-secretase state models. The density maps of the structures were generated with the molmap command of UCSF Chimera v1.11 (ref. 42) with a resolution of 8.0 Å.

## Results

We employed multiscale molecular dynamics (MD) simulations, combining coarse-grained (CG) and all-atom models, to investigate different conformational states of the γ-secretase complex. The initial models were derived from the available experimental information (PDB IDs: 5FN2 and ; 5FN3). Our initial all-atom models were simulated for 1 μs in order to relax the structure into a POPC lipid bilayer environment and derive secondary and tertiary structural information required to build valid CG models. Using the constructed CG model of the full enzyme complex in a POPC lipid bilayer, 50 independent 1 μs trajectories were simulated in order to thoroughly sample the dynamics on a time scale sufficient to observe critical large-scale structural transitions. Subsequently, all-atom models of the γ-secretase complex were constructed based on conformational distributions derived from the extensive CG simulations. Finally, the all-atom models of the bilayer-embedded γ-secretase complex were simulated for 100 ns each, in order to assess the overall behavior of the complex at the atomic scale.

In the following sections, we provide a detailed picture of (1) the relative roles of the enzyme subunits in stabilizing the structure of the **PS1** catalytic subunit, (2) the activation mechanism of the enzyme resulting from protonation of the catalytic aspartic acid residues, and (3) the characterization of the principal motions of **NCT** ECD related to transitions between two conformational states of the enzyme complex. Overall, this study provides the first complete picture of the γ-secretase complex and the relative role of the enzyme subunits in the activation mechanism.

### Protein dynamics and inter-subunit interactions of γ-secretase

γ-Secretase is a highly stable protein complex, the maturation and activation of which is related to the assembly of its four components.^18^ To develop a detailed atomistic description and evaluate the general dynamics of γ-secretase and its subunit interactions, we performed all-atom MD simulations of ; 5FN2 and ; 5FN3 derived models in a POPC lipid bilayer using the CHARMM36 force field (Fig. S1 and S2†). The initial structures display two important differences: (1) ; 5FN3 lacks the carboxyl terminal fragment of **PS1** TM6 (264–288) while ; 5FN2 contains the carboxyl terminal fragment of **PS1** TM6 and (2) ; 5FN3 presents a larger distance between the catalytic Asp residues (5.06 Å) compared to a shorter distance (3.89 Å) observed in ; 5FN2. Fig. 1B shows the all-atom fluctuation analysis of the ; 5FN2 structure model, depicting the high mobility of the **NCT** extracellular fragment and relatively low mobility of its TMs. **PEN-2**, TM5-7 of APH1, and TM2 of **PS1** exhibited higher mobility due to their exposure to lipids and fewer protein–protein contacts. In contrast, the **PS1** TMs in close contact with **PEN-2** and **APH-1A** exhibited low mobility and stable protein–protein interactions during the all-atom simulations. These findings are consistent with previous experimental observations suggesting that **PEN-2** and **APH-1A** play key roles in the catalytic subunit stabilization and activation.^43^ Our results also provide an explanation for the structural instabilities observed in an earlier simulation study of the isolated **PS1** subunit.^23^

Analysis of the apolar contributions to the inter-subunit binding free energies of the four γ-secretase components suggests that **PS1**-TM1, **PS1**-TM8 and **PS1**-TM9 are involved in critical interactions with **APH-1A**, contributing to the low observed energy values (Fig. S3A†). Similarly, we observed favorable interaction between **PS1**-TM4 and the first and third TMs of **PEN-2**. It is worth noting that we found significant interactions between the **PS1** TM3–TM4 loop and the N-terminal fragment of **PEN-2**, which was involved in significant contacts with a major lobe helix of **NCT**. This network of interactions may play an essential role in the communication pathway between these subunits and the **PS1** catalytic site. Fluctuation analysis reveals that the γ-secretase complex, including the catalytic subunit, remains structurally stable throughout the 1 μs all-atom simulations, consistent with previous experimental studies.^7,12^ An equivalent analysis of our ; 5FN3 model is shown in the ESI (Fig S1–S3†).

### Presenilin structural ensemble

To complement the atomic-scale fluctuation analysis, we explored in detail the structure, flexibility, and orientation of the **PS1** TMs in the POPC bilayer. Furthermore, we analyzed the dependence of the complex structure on the protonation state of the catalytic aspartic acid residues and the impact of protonation on the orientation of TMs defining the **PS1** structure in the active and inactive states of the enzyme.

#### **PS1**-TMs dynamics

The overall small changes in **PS1** structure observed during the all-atom simulations of the ; 5FN2 and ; 5FN3 derived models (Fig. S3B†) demonstrate that the initial structures of the complex remained stable throughout the simulations. Computed RMSF, which provides insight into residue mobility relative to the average simulation structure, was used to explore the TM mobility and loop flexibility in **PS1** (Fig. S3C†). The secondary structure of **PS1** was monitored and compared with the secondary structure derived from cryo-EM structural data demonstrating that the helicity of the TMs was preserved throughout the all-atom simulations (Fig. S3D†). The greatest flexibility was observed in loop regions separating relatively stable TMs. Due to the high flexibility of the TM1–TM2 loop (hydrophilic loop, HL1), Tomita and coworkers proposed that the HL1 loop may play a key role in substrate recognition.^44^ Similarly, Wolfe and coworkers proposed that HL1 together with the **PS1**-CTF, which comprise a large fragment of the extracellular/luminal side of **PS1**, contribute to the “initial” substrate-binding conformation before substrate reaches the **PS1** γ-site.^45^ Our simulation results support the plausibility of these conjectures.

**Fig. 2:**
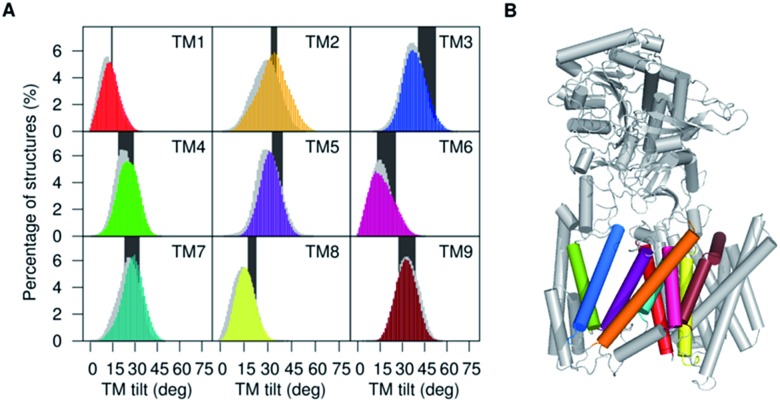
(A) Distribution of tilt angles of the **PS1** TMs calculated for simulations of ; 5FN2 (color coded by TM helix number) and ; 5FN3 (in gray shade) and compared with TMs tilt angle ranges obtained from available experimental structures of γ-secretase (PDB IDs: ; 5A63, ; 4UIS, ; 5FN2, ; 5FN3, ; 5FN4 and ; 5FN5) (black bars). (B) Depiction of the 3D structure of γ-secretase (color coding **PS1** TMs as (A)).

The TM6 cytosolic fragment also showed high flexibility during the MD simulations. It has been proposed that alterations in the distance between cytosolic sides of TM6 and TM7 correlate with Aβ42 production, suggesting that the TM6 conformation is a critical regulator of **PS1** catalytic activity.^46^ The large RMSF values and highly conserved helicity observed in TM2 and TM6 suggest the presence of collective motion modulating the relative orientation of these helices. The mobility of TM2 and TM6 has also been associated with the significant plasticity of the active site suggested by cryo-EM structural analysis.^12^ In Fig. 2 we present the computed tilt angle distribution of TMs, which agrees well with distributions derived from available γ-secretase structures. In particular, the wide tilt angle distribution of TM2 confirms the high mobility of this helix associated with the **PS1** subunit plasticity.

### **PS1** populates two principal conformational states

**PS1** is an aspartyl protease that contains two catalytic Asp residues located in the intramembrane regions of TM6 and TM7.^8,47^ Similar to other aspartyl proteases, the mechanism of catalysis is believed to involve formation of an initial intramolecular hydrogen-bond between the catalytic aspartic residues in coordination with a water molecule.^48,49^ In this acid–base mechanism, one Asp residue should be deprotonated (as aspartate, Asp^–^) to activate the water molecule, while the second Asp residue (as aspartic acid, Asp-H) donates a proton to the carbonyl group of the substrate.^50^ The initial hydrogen-bond formed between the aspartates is believed to be essential to the proton transfer step necessary to the overall enzymatic reaction.^51,52^ These observations suggest that the distance between the catalytic Asp residues should be less than 0.5 nm in order to form the crucial hydrogen-bond required for the formation of the **PS1** active state.^8^ Previous structural studies of γ-secretase indicate that the relative proximity and orientation of the catalytic residues in the **PS1** active site depend on the tilt angle orientation of different TMs of **PS1**.^12,18^ Inspired by this idea, large-scale conformational changes of γ-secretase were simulated for three coarse grained (CG) Martini models varying protonation states of the catalytic residues with either or both charged. Trajectories of 50 replicas were each simulated for 1 μs MD on each model. A p*K*_a_ calculation with the PROPKA v3.1 (ref. 53) module of PDB2PQR server^54^ provides estimates of Asp385 (p*K*_a_ = 9.91) and Asp257 (p*K*_a_ = 5.12), suggesting these residues should be protonated and unprotonated, respectively, at pH = 7.0. As a consequence of the many replicas used and long simulation time, we expected to observe no dependence on the initial conditions (Fig. S4†). In order to validate our Martini CG model, we simulated 5 μs of dynamics for a CG Martini model and a CG Martini model including an Elastic-Network in Dynamics (ElNeDyn) (Fig. S5 and S6†).^40^ The CG-ElNeDyn simulation exhibited smaller RMSD values (∼0.2 nm) throughout the simulation. However, the RMSF showed that our CG-ElNeDyn system restricted motion of the HL1 loop, the CTF of TM6, and the loop between TM8 and TM9 (PAL motif). Importantly, the distance between the catalytic Asp residues in the CG-ElNeDyn model remained practically fixed. As mentioned above, experimental studies show that mobility of these regions is essential to conformational changes in the complex required to form the active and inactive states of the catalytic subunit. It is important to note that despite these differences, measurements of key distances between the TMs led to similar results in 5 μs simulations of a Martini CG model and CG-ElNeDyn model. Finally, the curvature and kinks of the **PS1** TMs helices are consistent in all CG and AA simulation models (Fig. S7–S10†). Taken together these results validate our CG Martini models for the study of conformational changes of the γ-secretase enzyme complex.

Order parameters used to characterize the **PS1** ensemble include the TM tilt angles relative to the membrane normal (*T*_TM_) and the distance between the catalytic Asp (dd_Asp_). These order parameters are highly effective in differentiating the conformational state 1 (inactive state, long dd_Asp_ and proper *T*_TM_ angles) and state 2 (active state, short dd_Asp_ and proper *T*_TM_ angles) of γ-secretase. Short dd_Asp_ conformations involve Asp conformations suitable to form the crucial hydrogen bond required for the catalytic reaction.

**Fig. 3:**
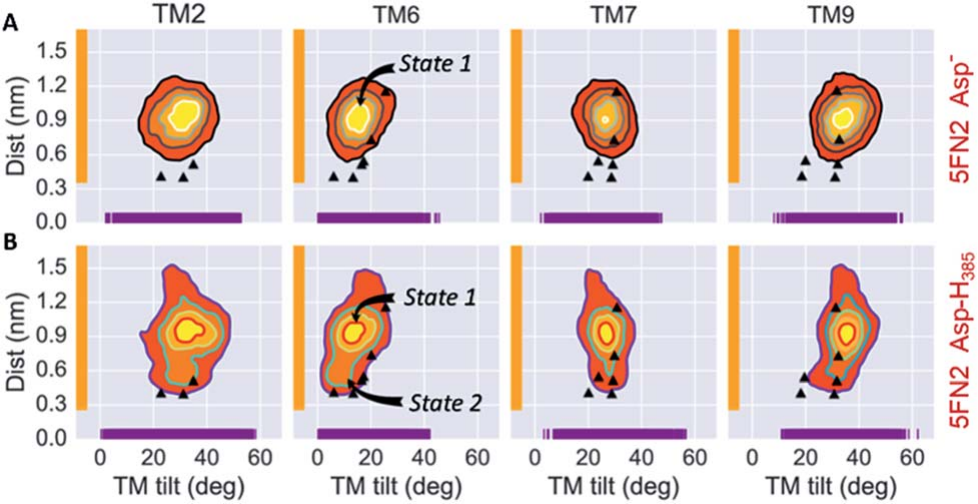
Simulated distributions of 5FN2 derived CG model in POPC bilayer projected onto (1) the distance between the catalytic residues (Asp257 and Asp385) and (2) the calculated TM2, TM6, TM7 and TM9 tilt angles in the (A) unprotonated and (B) Asp385 protonated states. The black triangles depict the values of dd_Asp_ and *T*_TM_ angles obtained from the experimental structures of γ-secretase (PDB IDs: ; 5A63, ; 4UIS, ; 5FN2, ; 5FN3, ; 5FN4 and ; 5FN5).

In order to explore the correlation between the TMs orientation and proximity of the catalytic residues, the set of 50 1 μs trajectories for the 5FN2 and ; 5FN3 derived CG models, varying Asp257 and Asp385 protonation states, were analyzed by projecting the simulated structural ensemble onto the dd_Asp_ distance and *T*_TM_ angles for TM2, TM6, TM7 and TM9. Fig. 3 displays the probability distributions for the dd_Asp_ distance and the *T*_TM_ angles for ; 5FN2 model of γ-secretase with unprotonated Asp and protonated Asp385 (similar results for ; 5FN3 and the two systems with the protonated Asp257 are available in Fig. S11†). There are notable changes in the relative probabilities of the conformational states of γ-secretase characterizing the structural ensemble of **PS1**: (1) the state 1, predominant in the unprotonated ; 5FN2 and ; 5FN3 derived CG models, is characterized by an inactive **PS1** catalytic conformation with a dd_Asp_ distance larger than 0.5 nm; (2) the state 2 observed in the protonated ; 5FN2 and ; 5FN3 derived CG models is characterized by a short dd_Asp_ distance consistent with active site formation. The *T*_TM_ angle distributions for the identified states indicate that TM6 and TM7 undergo an important *T*_TM_ angle modification that is strongly correlated with modulation of the dd_Asp_ distance. Moreover, the principal component analysis (PCA) of the **PS1** catalytic subunit suggests that a concerted motion of all TMs is required for the state 1 to state 2 transition (Fig. S12†).

Similarly, a slight change in the *T*_TM_ angles of TM2 and TM9 is observed to be correlated with a conformational change in *T*_TM_ angles of TM6 and TM7. Importantly, previous experimental studies have provided evidence that TM2 and TM9 constitute the initial substrate binding site in **PS1**, suggesting two different mechanisms of substrate entry into the active site: between TM6 and TM9 or between TM2 and TM6.^43,45^ Given the high flexibility and broad tilt angle distribution observed in our study, which is consistent with the conformational ensemble derived from cryo-EM studies, we specifically conjecture that TM2 acts as a gateway for substrate entry through TM2 and TM6.^55^

**Fig. 4:**
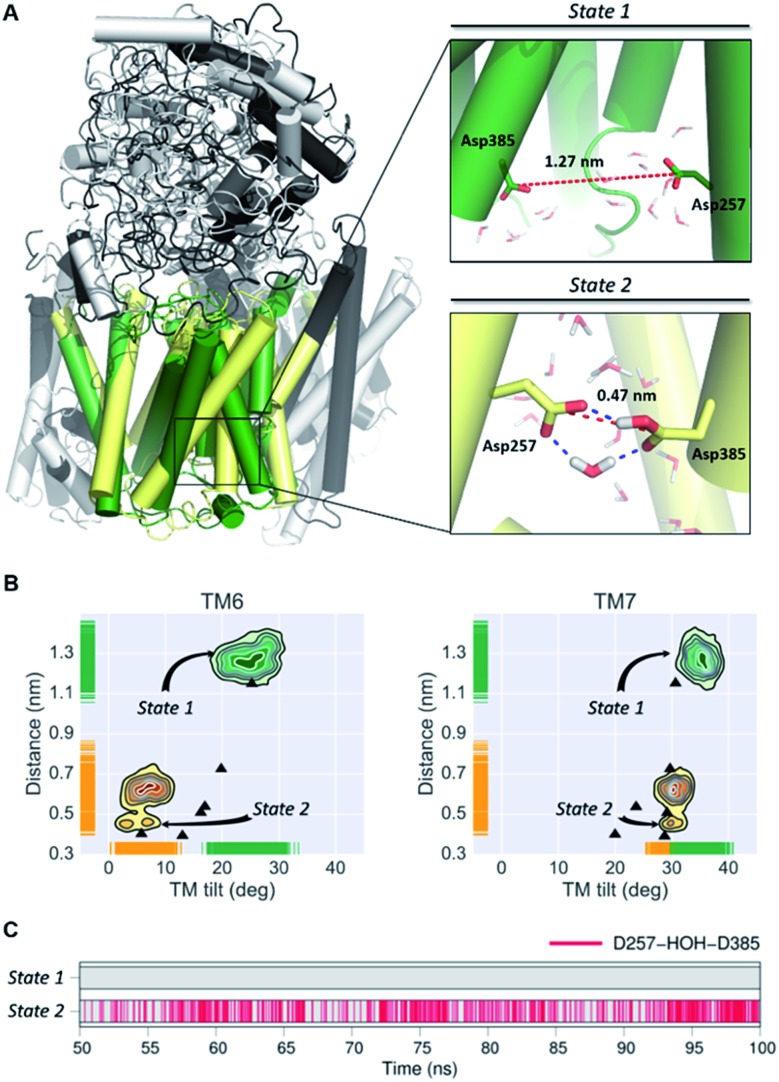
(A) Most representative structures of the state 1 (inactive) and state-2 (active) conformations of the **PS1** subunit of γ-secretase obtained from all-atom MD simulations. The dotted red line represents the distance between the gamma carbons of Asp257 and Asp385. (B) Simulated distribution of both conformational state models projected onto the distance between the catalytic aspartic acid residues and the **PS1** tilt angles of TM6 and TM7. The black triangles depict dd_Asp_ distances and *T*_TM_ angles obtained from the experimental structures of γ-secretase (PDB IDs: ; 5A63, ; 4UIS, ; 5FN2, ; 5FN3, ; 5FN4 and ; 5FN5). (C) Time evolution of hydrogen bonds between a coordinated water molecule and both catalytic aspartic residues through the last 50 ns of the state 1 and state 2 simulations.

In order to obtain a detailed atomistic description of the identified conformational states of **PS1** structures, we performed 100 ns all-atom MD simulations (Fig. 4) of the γ-secretase complex employing the CHARMM force field in a POPC lipid bilayer. The most probable state 1 and state 2 γ-secretase conformations were selected using the GROMACS cluster tool with a RMSD threshold of 2.0 Å from the CG structural ensemble. As observed in the CG studies, the state 2 conformation is showed to fluctuate between more and less active conformations (dd_Asp_ distance does not exceed 0.75 nm). This state 2 (active/less-active) equilibrium is associated with Asp dihedral angle fluctuation with unaltered inclination of the key TMs. Equivalent fluctuations between state 1/state 2 (inactive/active) states were observed in the CG model simulations, indicating that the state 2 (active) conformation may require the substrate to be bound to the catalytic site in order to stabilize the active conformation. On the other hand, data derived from atomistic MD simulation of the **PS1** state 1 (inactive) conformations display substantial structural fluctuations with a 1.0 nm dd_Asp_ distance. This observation is consistent with the previously characterized inactive CG simulations. Furthermore, the analysis of water molecules at the active site showed the presence of intramolecular hydrogen-bonds between both catalytic aspartic residues and a coordinated water molecule within 3.0 Å distance and 150-degree angle (Fig. 4C). This hydrogen bond network was present 43.92% of the time of the last 50 ns of the state 2 all-atom simulation. Interestingly, this water coordination is a crucial step for **PS1** activation and proteolytic processing of γ-secretase substrates.

### Nicastrin shows dramatic up-down movement and left-right rotation of the **NCT** extracellular domain

The large conformational modifications observed during cryo-EM studies of the complex have led to several hypotheses that seek to define the substrate recognition mechanism of γ-secretase.^20,22,56^ The observed structural changes in γ-secretase complex can be related to the large-scale motion of the **NCT** extracellular domain. Employing the 50 1 μs CG MD simulations of our ; 5FN2 and ; 5FN3 derived models, we performed PCA to identify the most significant collective motions of the complex, for both protonated and unprotonated states. The PCA analysis was performed with the GROMACS inbuilt tools (covar and anaeig).^30^

**Fig. 5:**
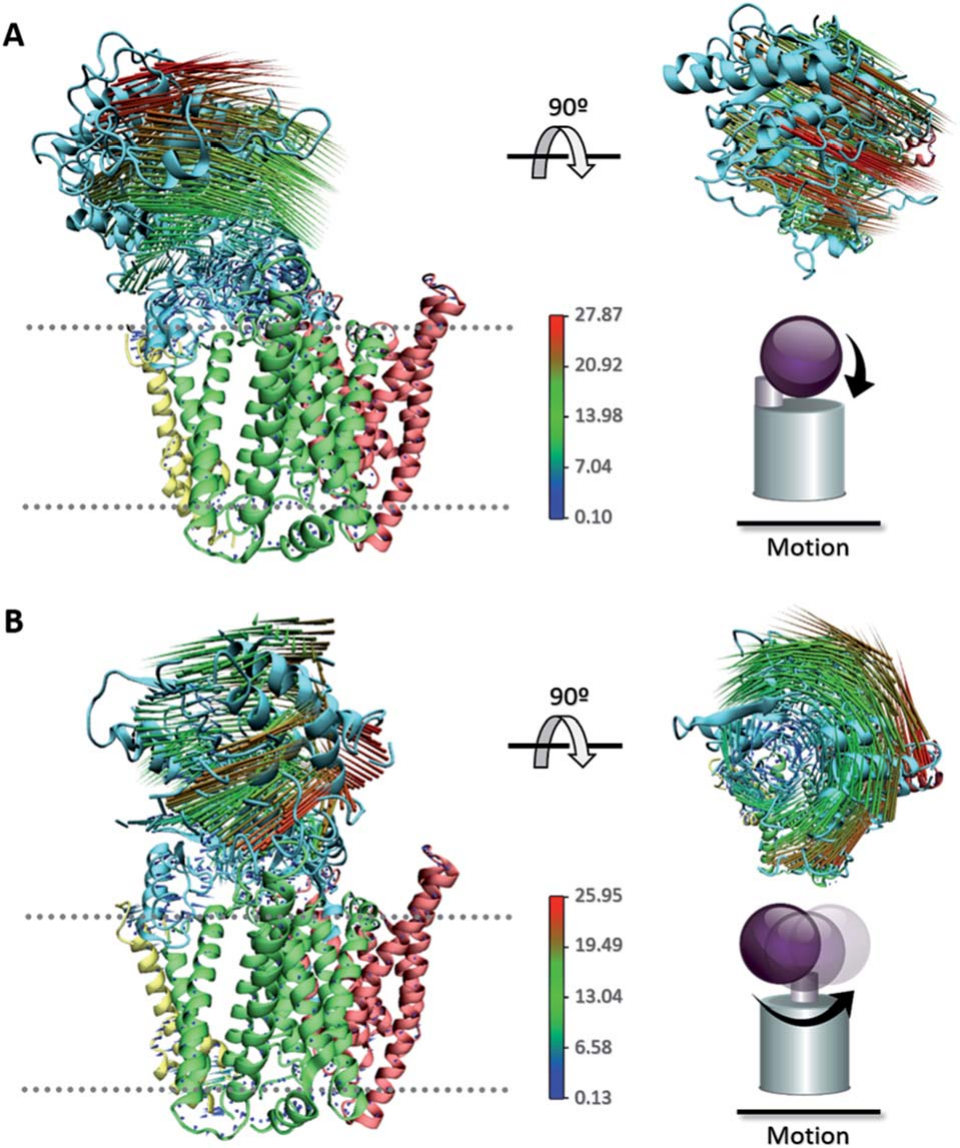
Porcupine representation of the (A) “up/down” movement and (B) “left/right” rotation of the **NCT** ECD obtained from PCA.

The first two eigenvectors, involving the **NCT** ECD and accounting for the majority (23%) of overall γ-secretase motion, were selected to analyze the **NCT** movement. Analysis of conformations projected onto the first two PC eigenvectors revealed that the first eigenvector is related to **NCT** “up/down” movement while the second eigenvector corresponds to **NCT**-ECD “left/right” rotation. Fig. 5 depicts motion along the first and second principal components (PCs) using a porcupine representation. Interestingly, we found the same two principal component motions during the 5 μs simulation of our CG-ElNeDyn model with a slower dynamics and a smaller set of collective vectors (Fig. S6†).

In order to further analyze structural fluctuations of **NCT**, we constructed two order parameters based on the first and second PCs obtained from the CG structural ensemble (capturing up/down and left/right rotational motion of **NCT**, Fig. S13†). The first order parameter describes the distance between the center of geometry of **PS1** and the center of geometry of the large lobe of **NCT** (dd_PS-NCT_). Significant variation in the distribution of the dd_PS-NCT_ distances indicates that a percentage of the γ-secretase structures simulated using the CG model exhibit an “up” conformation (with dd_PS-NCT_ distances larger to 5.0 nm) while a fraction of the **NCT** domain adopts a “down” conformation (with a dd_PS-NCT_ distance shorter than 4.5 nm). The second order parameter characterizes the rotational motion of **NCT** (dih_NCT_) and involves a dihedral angle formed by four points of the NCT structure: (1) the intracellular and (2) extracellular amino acids of the single TM helix of **NCT**, (3) the center of mass of the large lobe of **NCT**, and (4) the residues Arg583–Asp588 (located at the distal end of the **NCT** large lobe). Previous experimental structural studies of γ-secretase have proposed a possible interaction of the substrate N-terminal region with exposed Glu333 located at a lid opening.^17^ Taken together, those observations and our simulation analysis support the conjecture that **NCT** rotation (dih_NCT_) is essential to the γ-secretase substrate binding mechanism. The simulated dih_NCT_ distribution indicates that the **NCT** large lobe rotates in the left/right directions, with preference for leftward rotation (dih_NCT_ values of –50 deg). However, further analysis demonstrates that the identified **NCT** motion, described by the dd_PS-NCT_ distance and the dih_NCT_ rotation, does not modify the position of the **NCT**-lid (Fig. S14 and S15†). This suggests that Glu333 might not be involved in the substrate recognition mechanism. Furthermore, Wolfe and coworkers^57^ proposed that the **NCT**-ECD acts as a steric gatekeeper for substrate entry into the **PS1** active site, instead of having a specific interaction with the substrate. This steric block mechanism could be associated with ECD up-down movement, left-right rotation, and interaction with the transmembrane subunits, as shown in the following section.

#### The structural ensemble of γ-secretase is characterized by three unique conformational states

**Fig. 6:**
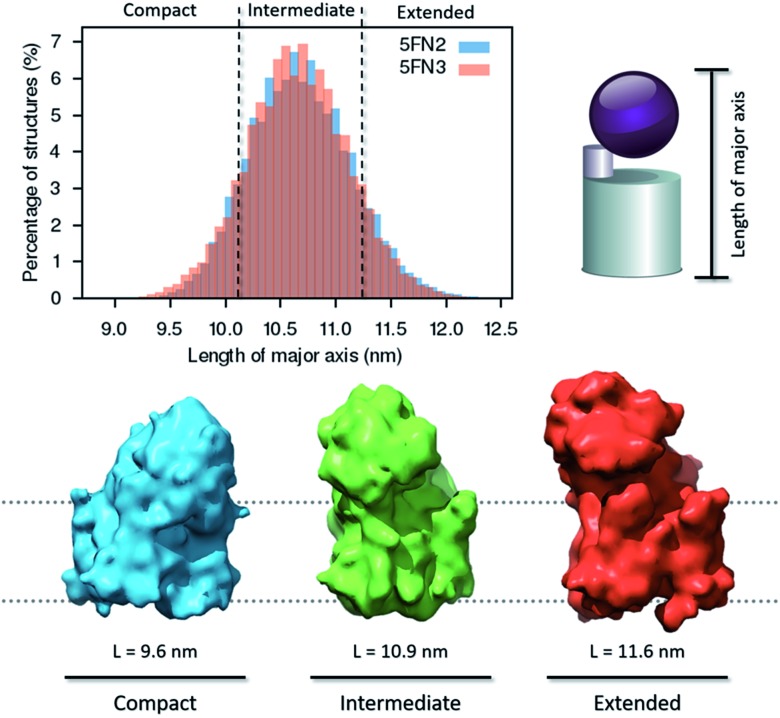
Distribution of major axis length and density map representation of the compact, intermediate, and extended conformations of the γ-secretase complex derived from 50 1 μs CG replica simulations of the γ-secretase complex.

The simulated γ-secretase structural ensemble was compared with the experimentally derived conformational distribution of the γ-secretase complex.^22^ We measured the major axis length of our structures using the distance between the amino acids located at the lower intracellular and upper extracellular domains (Fig. 6).^22^ In an experimental single-particle EM study, Chávez-Gutiérrez and coworkers^22^ found that wild-type γ-secretase exists in three different structural states: compact, intermediate, and extended conformations. In agreement with their experimental observations, three structural states of the γ-secretase complex were identified in the simulated length distribution. A dynamic equilibrium was observed, characterized by a greater abundance of the intermediate form. On the other hand, our CG-ElNeDyn simulation model only sampled the extended and intermediate groups due to restrictions imposed by the elastic network (Fig. S6C†).

In further analysis, we extracted representative structures characterizing the three distinct conformational states in order to generate and analyze three density maps (8.0 Å resolution), employing UCSF Chimera software.^42^ The structural state transitions are observed to involve three distinctive interactions between the extracellular domain of **NCT** and key γ-secretase subunits: (1) in the extended state, the small lobe of **NCT** interacts with the C-terminal region of **PEN2**, (2) in the intermediate state, the large lobe of **NCT** interacts with the HL1 (hydrophilic TM1–TM2 loop) of **PS1**, and (3) in the compact state, the large lobe of **NCT** interacts with the **APH-1A** and **PS1** extracellular domains. The broad distribution of structures in the intermediate state indicates that the second interaction, between the large lobe of **NCT** and HL1 of **PS1**, is essential for **NCT** ECD stabilization. Moreover, this conformation could be essential for **NCT** to act as a selective “gatekeeper” during peptide entry into the catalytic site.^58^ Experimental studies have demonstrated that when substrate or other agents (*e.g.* inhibitors and modulators) are bound to γ-secretase, the compact conformation of the complex is the most stable and favored state.^20,22,56^ Importantly, we found that although the **NCT** ECD interact with the hydrophilic **PS1** HL1, **APH-1A**, and **PEN2**, no correlated motion was observed between the large **NCT** ECD and the **PS1** TMs helices. These results suggest that the large **NCT** ECD motion is not critical to the activation or inactivation mechanism of the catalytic **PS1** subunit (Fig. S16†).

## Conclusions

Our multiscale simulation approach has elucidated the first detailed atomistic description of the structure and dynamics of the complete γ-secretase complex. Analysis of the structure and dynamics of our simulated γ-secretase models supports the hypothesis that **PEN-2** and **APH-1A** play essential roles in stabilizing the **PS1** catalytic subunit through interaction with the **PS1** N-terminal and C-terminal fragments, respectively.

Overall, the TMs of γ-secretase form a stable structural complex of relatively low mobility. However, we specifically observed that higher flexibility and correlated motion of **PS1**-TM2 and **PS1**-TM6 impacts the dd_Asp_ distance essential for protease catalytic activity. Taken together, these findings suggest that correlated motion of key TMs helices, dependent on the dd_Asp_ distance and TM tilt angles, is essential to the transitions between inactive to active states of **PS1**.

We have further demonstrated that the equilibrium between the state 1 (“inactive”) and state 2 (“active”) **PS1** conformers is modulated by the protonation states of the catalytic residues Asp257 and Asp385. State 1 **PS1** conformations are predominant in proteins having unprotonated catalytic Asp residues, while state 2 **PS1** conformations are sampled when either catalytic Asp residue is protonated.

Analysis of global conformational changes in the γ-secretase complex identified significant mobility in the **NCT** ECD, characterized as up/down motion and left/right rotation of the large lobe of **NCT** ECD. Similar movement of the **NCT** ECD has been previously inferred from electron microscopy images.^22^ Based on these observations, it has been conjectured that γ-secretase function is controlled through modulation of relative populations of **NCT** ECD conformational states in the γ-secretase structural ensemble. Importantly, our data suggests that these movements are not correlated with **NCT** lid motion and the state 1/state 2 equilibrium of **PS1**, supporting that **NCT**-ECD only acts as steric gatekeeper for substrate entry into the active site, as proposed first by Wolfe and coworkers.^57^

This multiscale simulation analysis provides a detailed picture of the global structure and dynamics of the γ-secretase complex. The insight provided into the nature of the active and inactive state conformation, as well as the mechanism of transition between state 1 and state 2 of the **PS1** catalytic domain, provides a foundation for future studies of the catalytic mechanism of substrate recognition and cleavage. It is our hope that the results of this study will contribute to a mechanistic understanding of the cleavage of APP-C99 by γ-secretase in the genesis of Aβ, critical to the structure-based design of AD therapeutics.

## Conflict of interest

The authors declare no competing financial interest.
