# Supplementary material for: Characterizing the structural ensemble of γ-secretase using a multiscale molecular dynamics approach
Source: Chem Sci. 2017 Jun 5;8(8):5576–84. doi: 10.1039/c7sc00980a (PMC5618787; doi:10.1039/c7sc00980a)
Supplement: Supplementary file 1 [file SC-008-C7SC00980A-s001.pdf]

Electronic Supplementary Material (ESI) for Chemical Science.
This journal is © The Royal Society of Chemistry 2017



# Characterizing the structural ensemble of γ-secretase using a multiscale molecular dynamics approach

Rodrigo Aguayo-Ortiz[a], Cecilia Chávez-García[a], John E. Straub[b], and Laura Dominguez[a,*]

[a]*Departamento de Fisicoquímica, Facultad de Química, Universidad Nacional Autónoma de México, Mexico City, 04510, Mexico;* [b]*Department of Chemistry, Boston University, Boston, Massachusetts 02215, United States*

***Corresponding Author:** Phone: +52-5622-3773. E-mail: lauradd@unam.mx

*Table of contents*


| Contents | | Page |
|---|---|---|
| **Fig. S1** | RMSD of 5FN2 and 5FN3 derived all atom model components | 1 |
| **Fig. S2** | RMSF of 5FN2 and 5FN3 derived all atom model components | 2 |
| **Fig. S3** | Presenilin contribution energy, RMSD, RMSF and % helicity (%) for all-atom MD simulations. | 3 |
| **Fig. S4** | Projection of the Asp257-Asp385 distance and TM helix tilt of the CG models | 4 |
| **Fig. S5** | Time evolution of RMSD, RMSF, and key PS1 TMs distances calculated for 5 μs of CG-Martini and CG-ElNeDyn systems. | 5 |
| **Fig. S6** | PCA of the NCT ECD for the 5 μs simulation of the CG-Martini and CG-ElNeDyn systems as well as the distribution of the major axis length of the complex | 6 |
| **Fig. S7** | Curvature of the PS1 TMs during the last 500 ns of the 50 CG replicas | 7 |
| **Fig. S8** | Measurement of the per-residue kink angle for each PS1 TM of the 5FN2-derived models | 8 |
| **Fig. S9** | Measurement of the per-residue kink angle for each PS1 TM of the 5FN3-derived models | 9 |
| **Fig. S10** | Schematic representation of the kink angles of the 5FN2 PS1-derived AA and CG models | 10 |
| **Fig. S11** | Time-colored (ns) projection of the Asp257-Asp385 distance and TM helix tilt of the Asp257 protonated system | 11 |
| **Fig. S12** | PCA of the 5FN2 PS1-derived AA and CG models and the porcupine representation of PC1 | 12 |
| **Fig. S13** | Simulated distribution for CG models projected onto $dd_{PS\text{-}NCT}$ distance and $dih_{NCT}$ rotation | 13 |
| **Fig. S14** | Projection of NCT lid and major lobe distance and NCT rotation | 13 |
| **Fig. S15** | Projection of NCT lid and Glu333 distance and NCT rotation | 14 |
| **Fig. S16** | Projection of γ-secretase *state 1* and *state 2* conformations and length of the major axis | 14 |

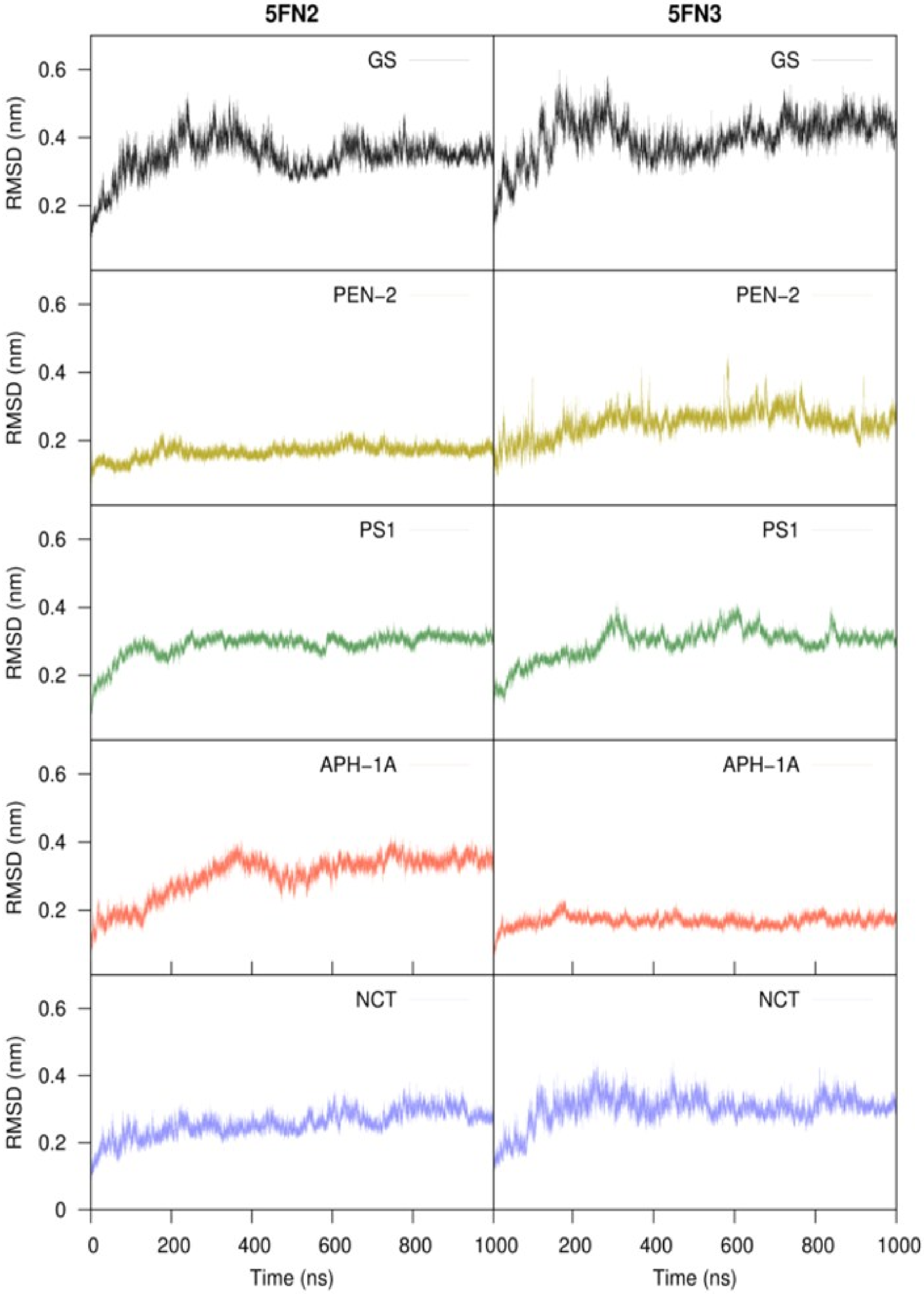


**Fig. S1.** Root mean square deviation (RMSD) of 5FN2 and 5FN3 derived all atom models of γ-secretase (GS) components: presenilin enhancer 2 (PEN-2), presenilin 1 (PS1), anterior pharynx-defective 1A (APH-1A), and Nicastrin (NCT).

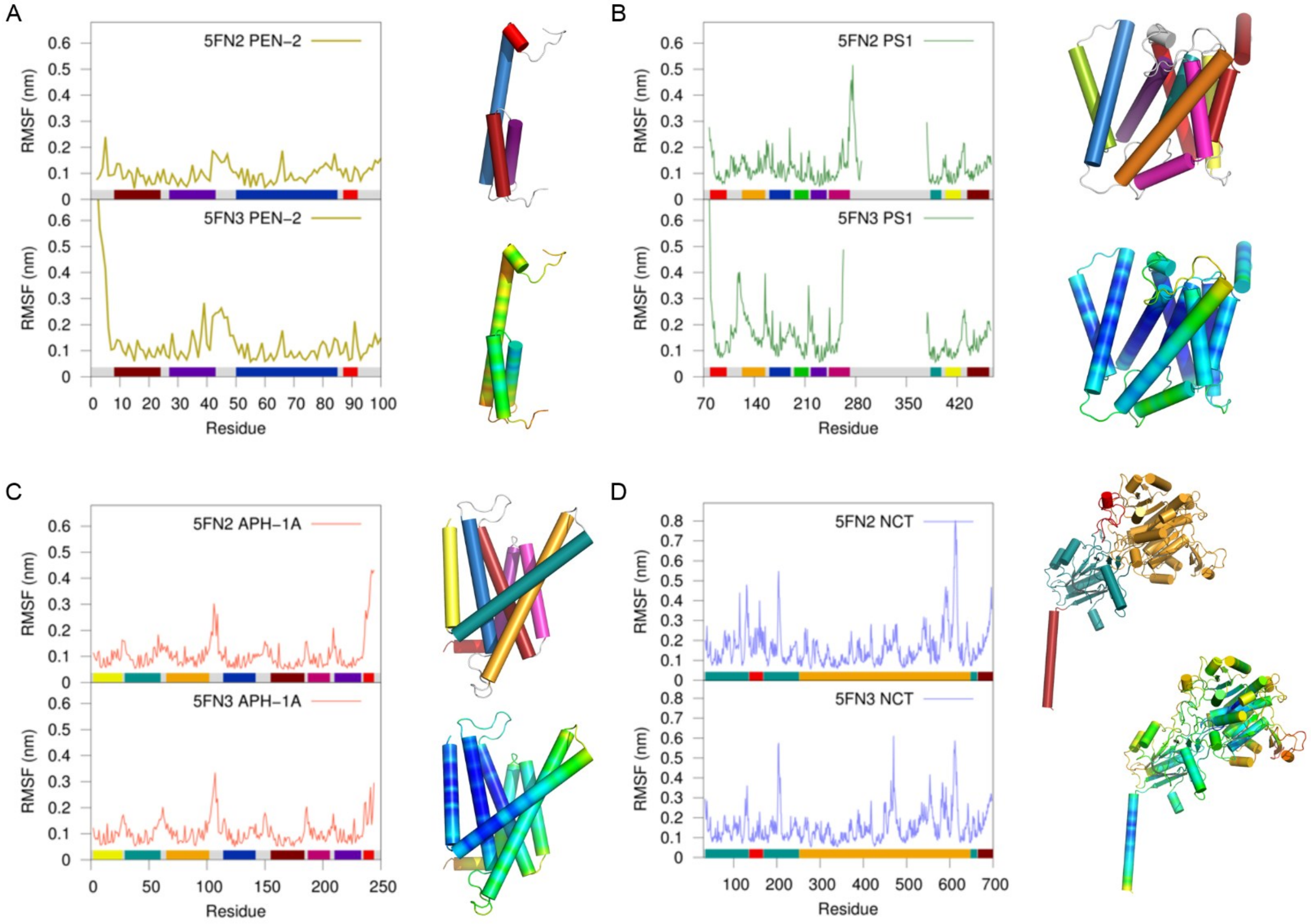


**Fig. S2.** Root mean square fluctuation (RMSF) of 5FN2 and 5FN3 derived all-atom models: (A) PEN-2, (B) PS1, (C) APH-1A and (D) NCT. The 3D structures depict the region represented in the RMSF plots (top) and the normalized per-residue RMSF (bottom) colored by the RMSF value from more flexible (red) to less flexible (blue).

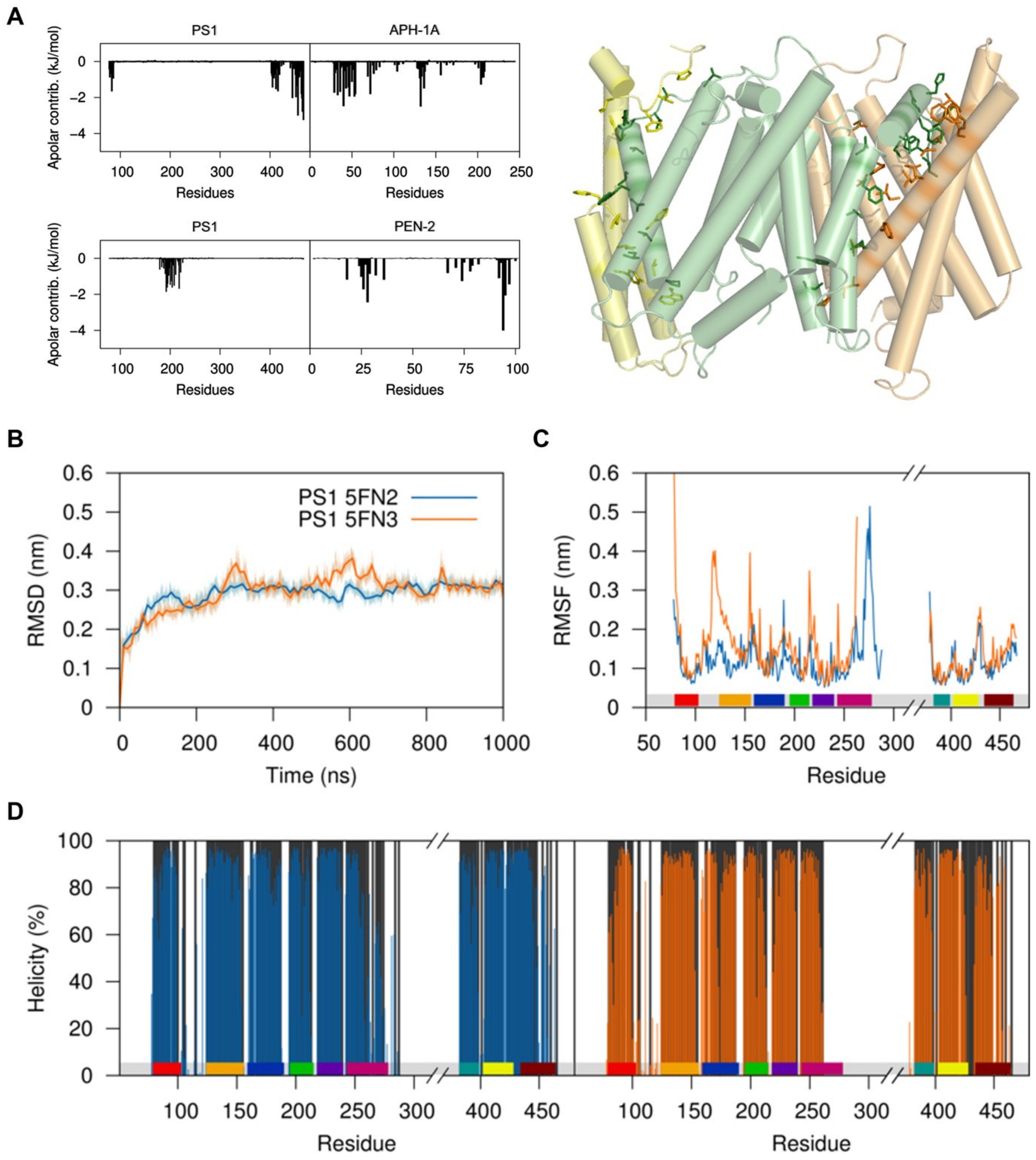


**Fig. S3.** (A) Calculated apolar free energy contribution of each amino acid by domain. Key residues making dominant contributions to apolar binding free energy are shown (color coded as in Figure 1A). (B) PS1 backbone root mean-square deviation (RMSD) during all-atom MD simulations of the 5FN2 (blue) and 5FN3 (orange) derived γ-secretase complex. The equilibrium states of the systems were reached after 300 ns of simulation, with resulting structures within approximately 0.30 nm RMSD of the initial structure. (C) Root mean-square fluctuation (RMSF) and (D) percent helicity of residues constituting the PS1 TMs.

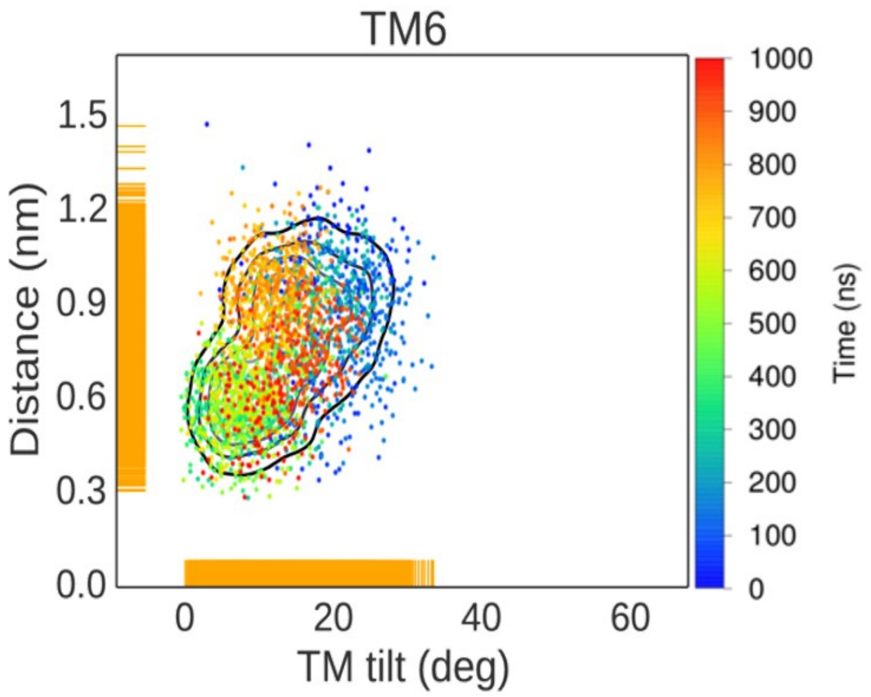


**Fig. S4.** Time evolution of one replica (out of fifty) of a CG 5FN2 derived model in a POPC bilayer projected onto the distance between the catalytic residues (Asp257 and Asp385) and the calculated TM6 tilt angle in the Asp257 protonated state, colored by time.

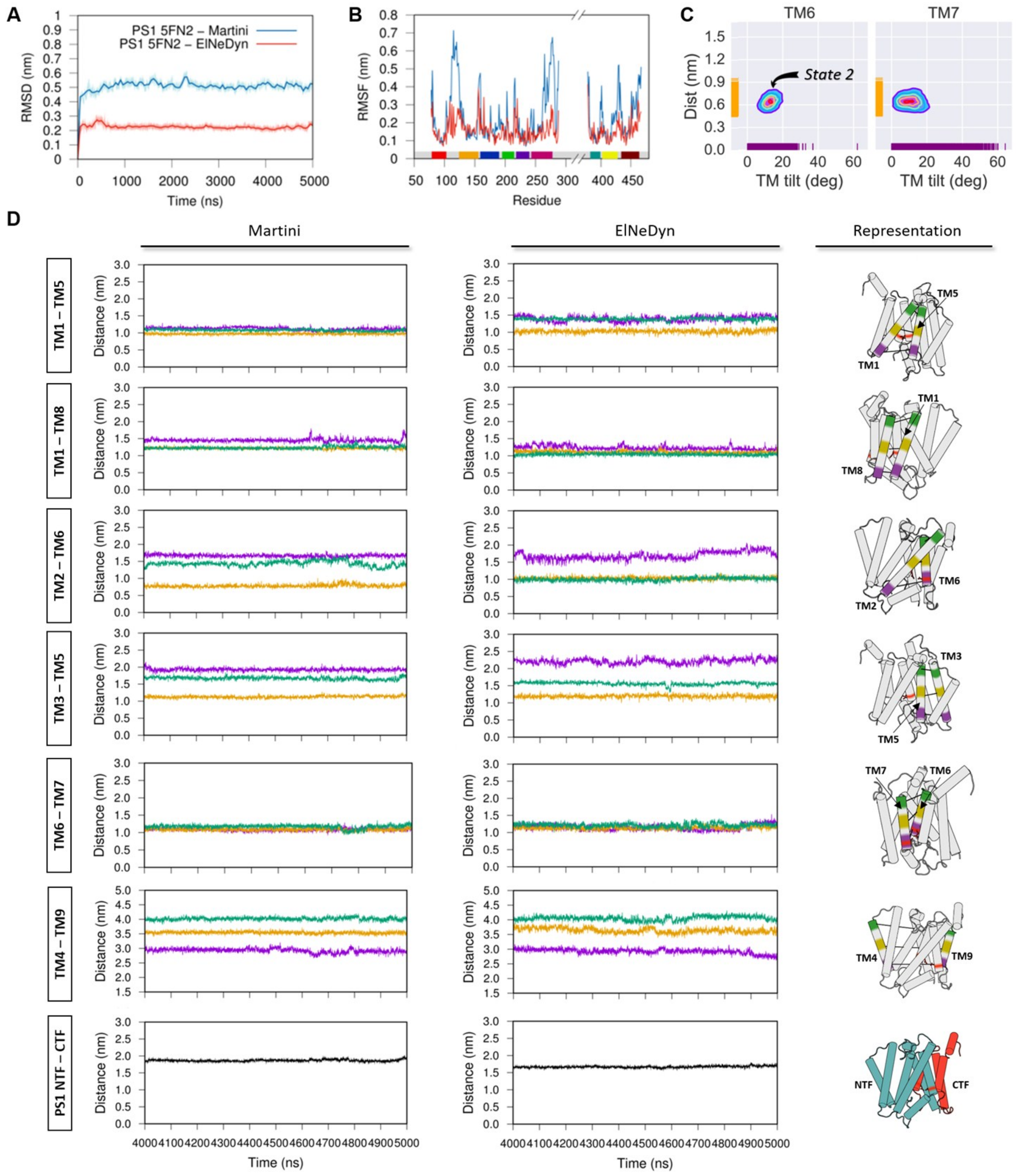


**Fig. S5.** (A) RMSD and (B) RMSF of the 5 µs CG Martini and CG-ElNeDyn PS1 derived models in a POPC bilayer. (C) Simulated distributions of our Martini CG-ElNeDyn 5FN2-derived simulation model in a POPC bilayer projected onto (1) the distance between the catalytic residues (Asp257 and Asp385) and (2) the calculated TM6 and TM7 tilt angles for the unprotonated state. (D) Time evolution of six key TM-TM distances obtained from the catalytic PS1 subunit during the last µs of the 5 µs 5FN2-derived CG Martini model trajectory and the last µs of the 5 µs 5FN2-derived CG-ElNeDyn model trajectory, together with a PS1 3D model to depict the measured distances and the color code segments in the plot. The last plot (PS1 NTF-CTF) depicts the distance between the centers of geometry of the PS1 C-terminal (CTF red) and N-terminal (NTF blue) fragments.

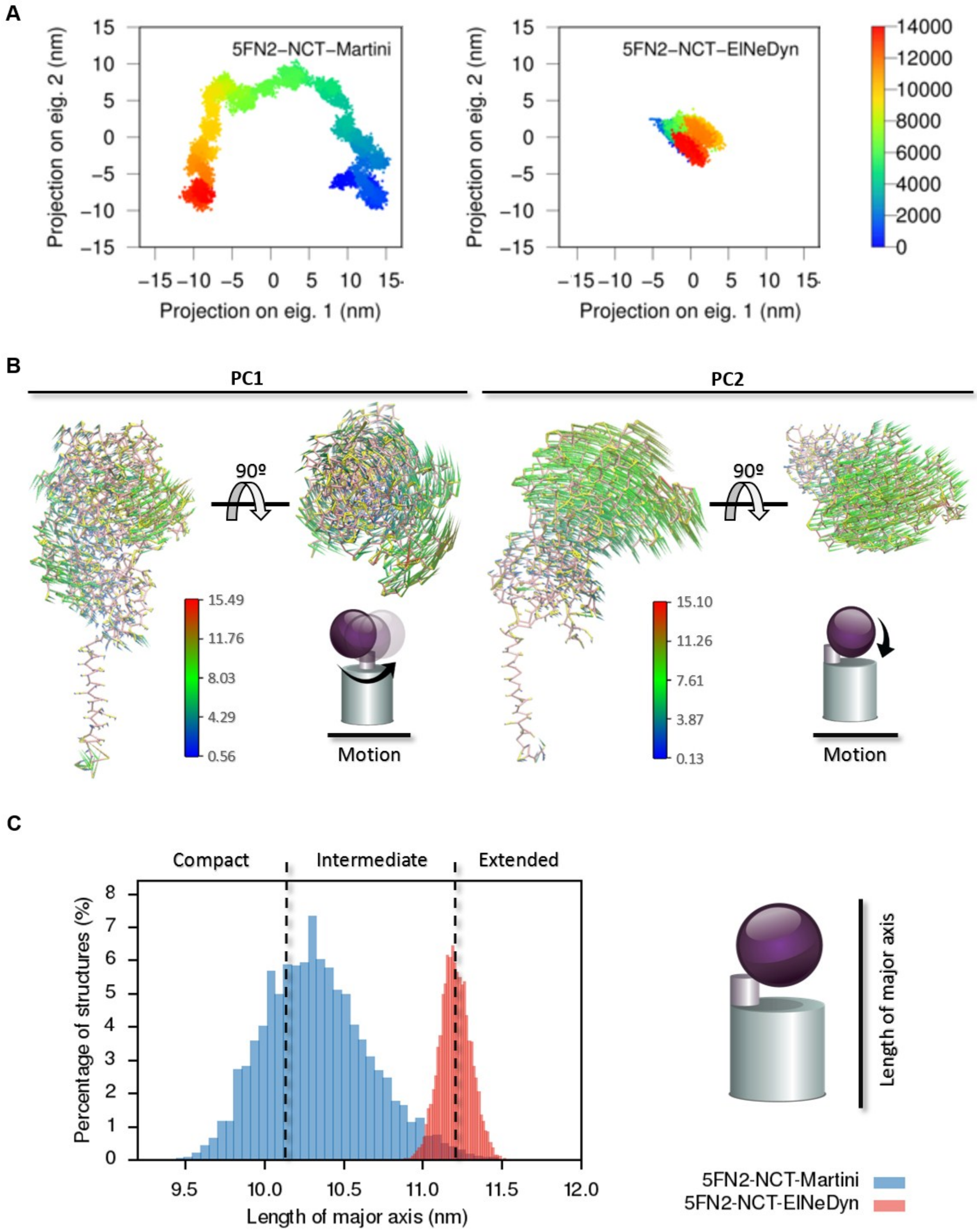


**Fig. S6.** (A) Projection of the first and second eigenvectors obtained from the PCA of the last 500 ns of the Martini CG trajectory (left) and the projection of the 5FN2 CG-ElNeDyn trajectory into the same first and second eigenvectors derived from the Martini CG trajectory (right). (B) Porcupine representation of the NCT ECD "left/right" rotation (PC1) and "up/down" movement (PC2) obtained from the PCA of CG-ElNeDyn 5FN2-derived model. (C) Distribution of major axis length of the compact, intermediate, and extended conformations of the γ-secretase complex derived from 5 μs Martini CG-ElNeDyn model simulations.

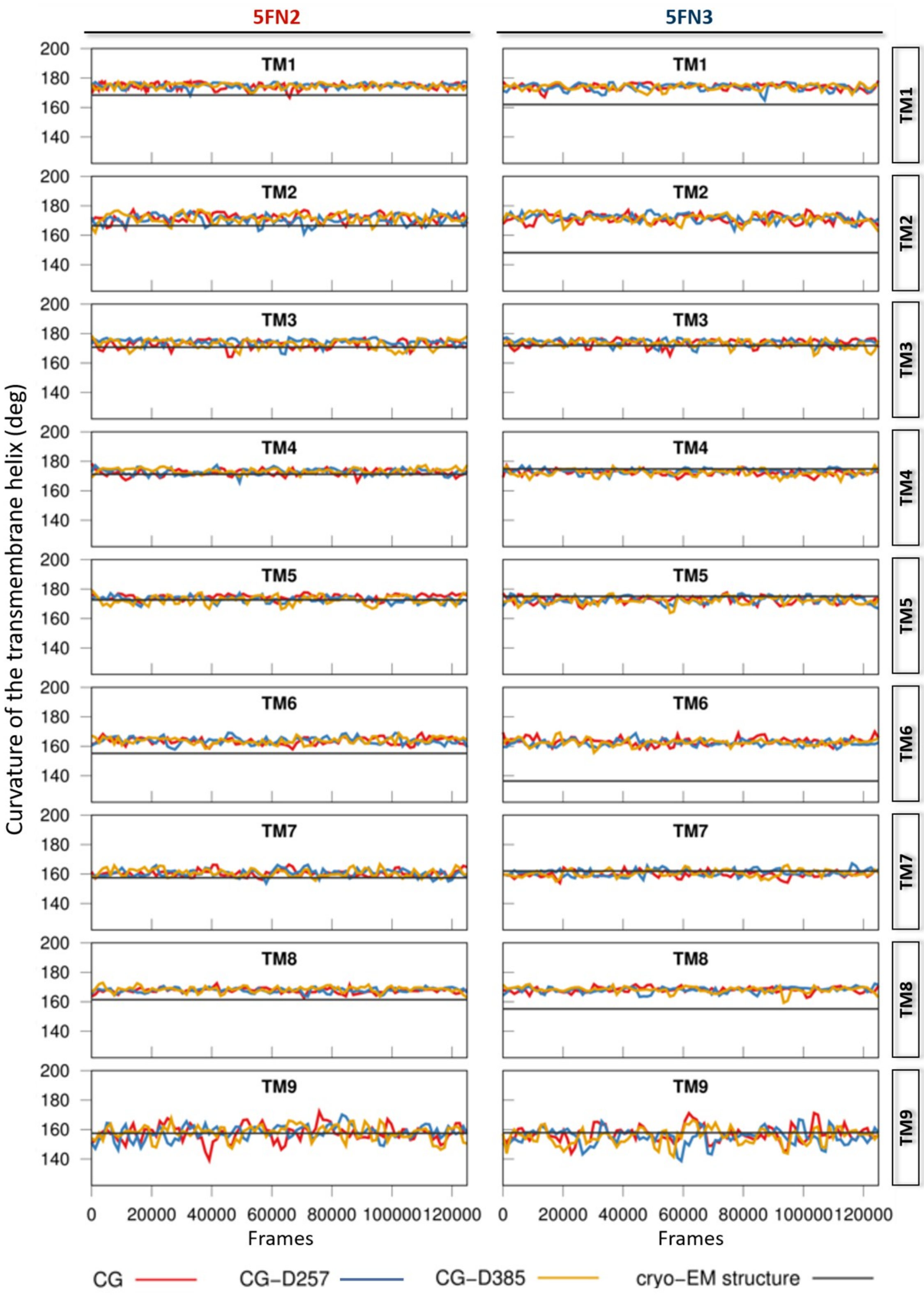


**Fig. S7.** PS1 TMs helix curvatures during the last 500 ns of the 50 CG replicas (125,000 frames) of the distinct γ-secretase 5FN2-derived (left) and 5FN3-derived (right) models in the unprotonated state (red) and the protonated D257 state (blue) and protonated D385 state (yellow) models, together with the reference curvature value for the cryo-EM structure (grey).

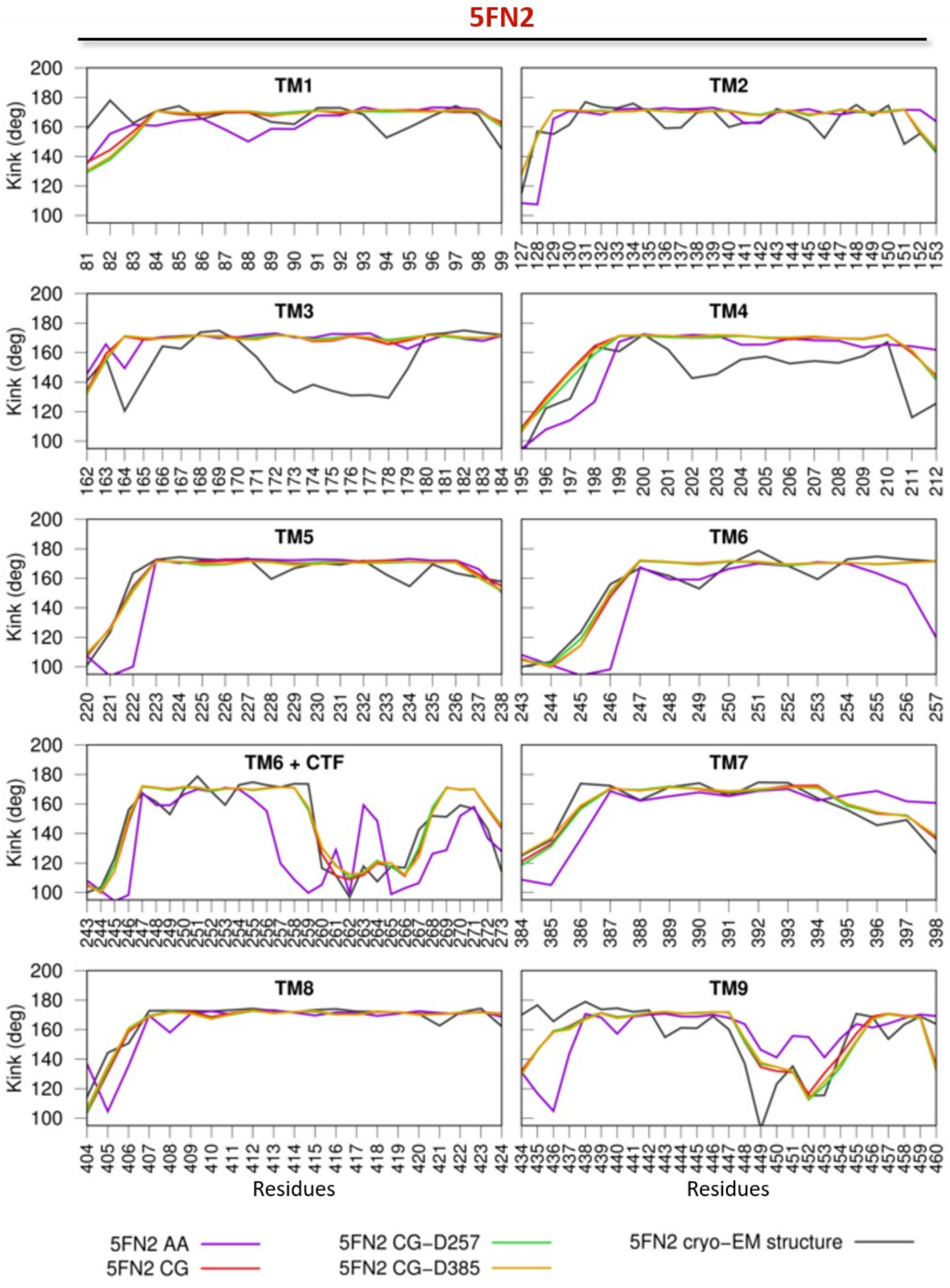


**Fig. S8.** Per-residue kink angles calculated for each PS1 helix in the AA (dark-violet), CG (red), protonated CG-D257 (green), and protonated CG-D385 5FN2-derived simulation models compared with the per-residue 5FN2-derived cryo-EM structure reference value (grey).

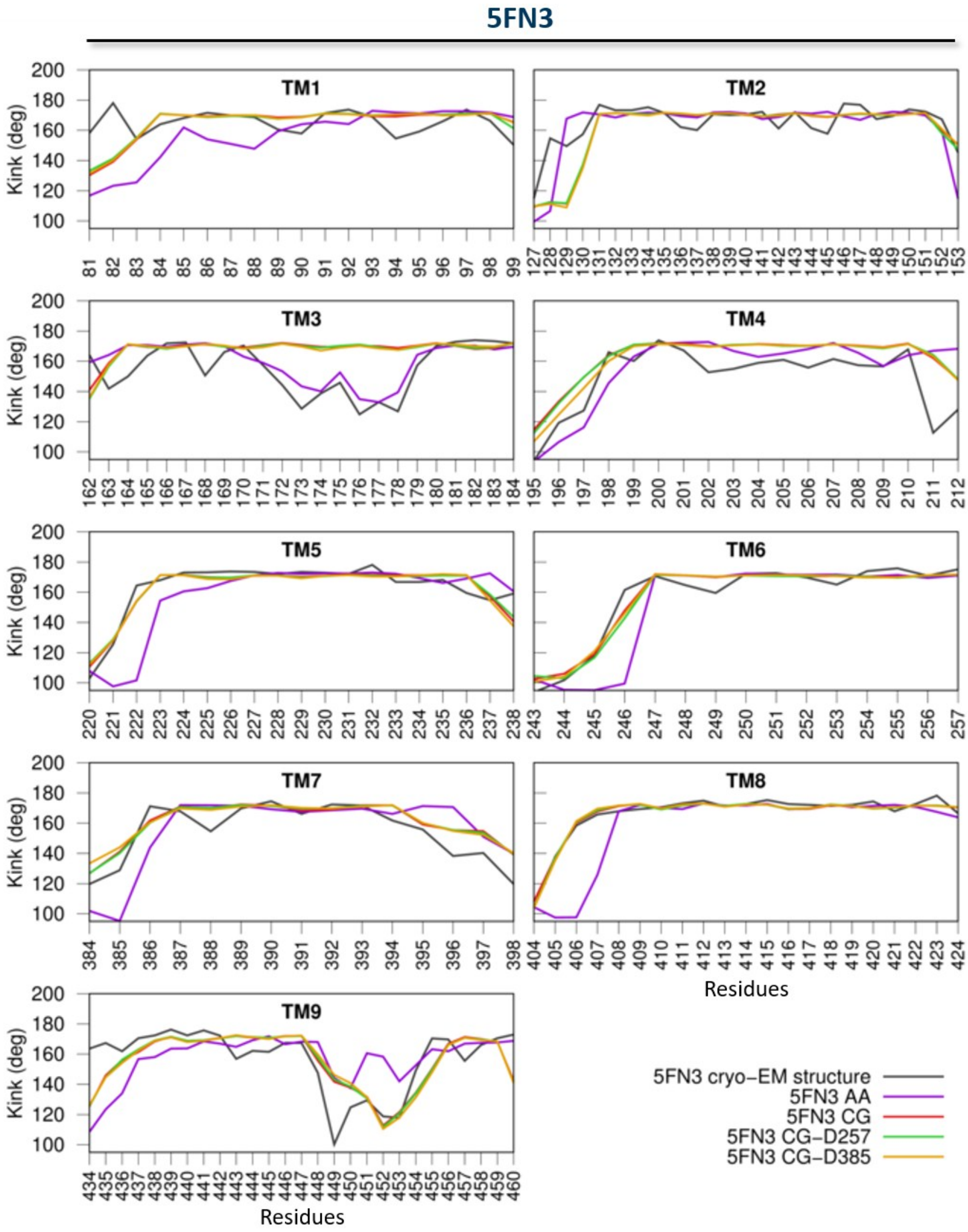


**Fig. S9.** Per-residue kink angles calculated for each PS1 TM helix in the AA (dark-violet), CG (red), protonated CG-D257 (green), and protonated CG-D385 5FN3-derived simulation models compared with the per-residue 5FN3 cryo-EM structure reference value (grey).

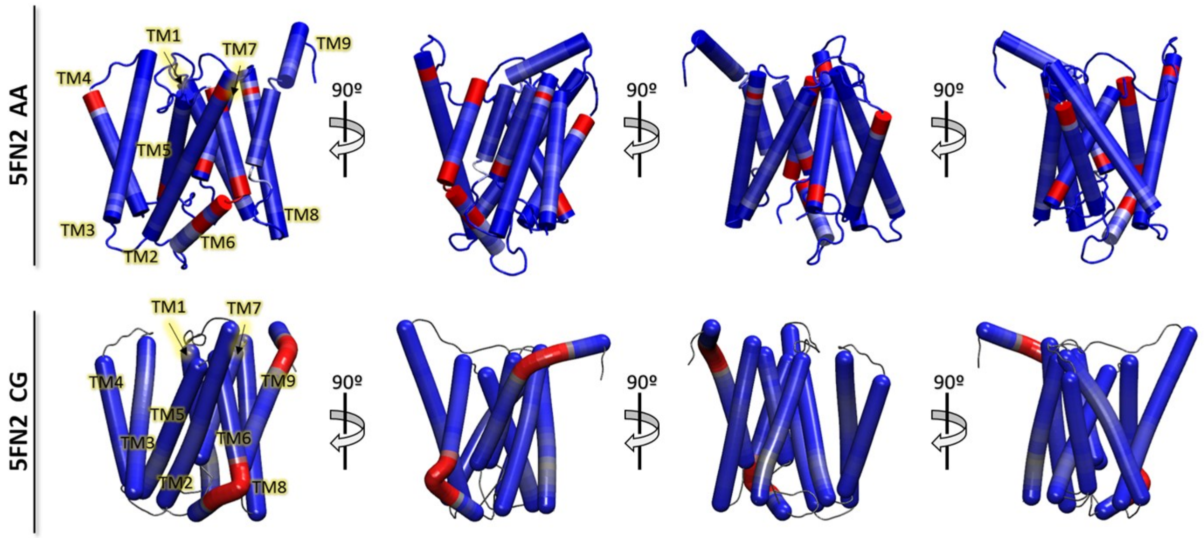


**Fig. S10.** Schematic representations of 5FN2 PS1-derived atomistic (top) and CG Martini (bottom) models of γ-secretase color-coded by our calculated kink angles, from more kinked (red) to less kinked (blue) values. The CG Martini model representation was generated with Bendix.

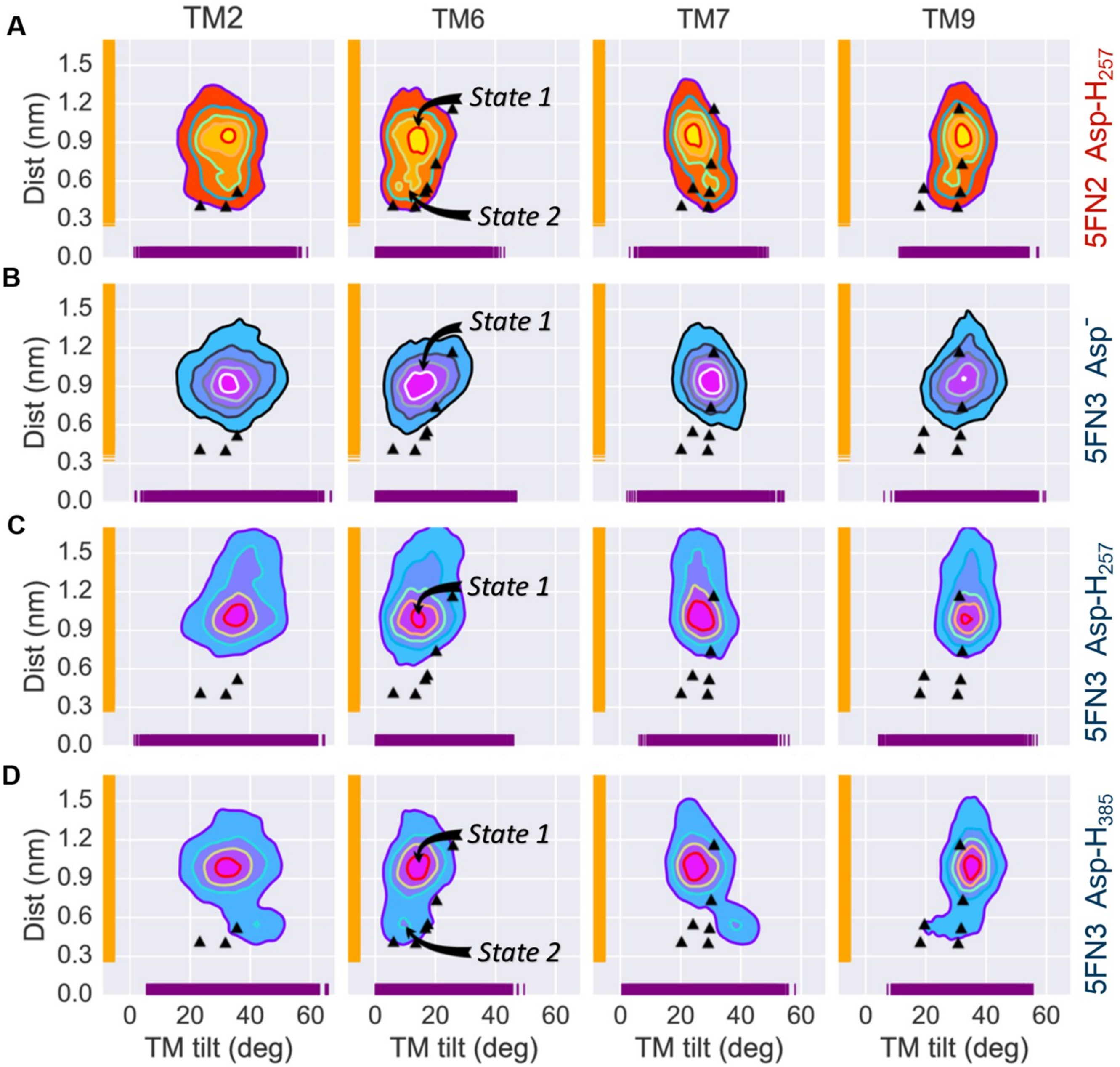


**Fig. S11.** Simulated distributions of 5FN2 (orange) and 5FN3 (blue) derived CG models in a POPC bilayer projected onto (1) the distance between the catalytic residues (Asp257 and Asp385) and (2) the calculated TM2, TM6, TM7 and TM9 tilt angles in the unprotonated and Asp257 and Asp385 protonated states. The black triangles depict the values of $dd_{Asp}$ and $T_{TM}$ angle obtained from the experimental structures of γ-secretase (PDB IDs: 5A63, 4UIS, 5FN2, 5FN3, 5FN4 and 5FN5).

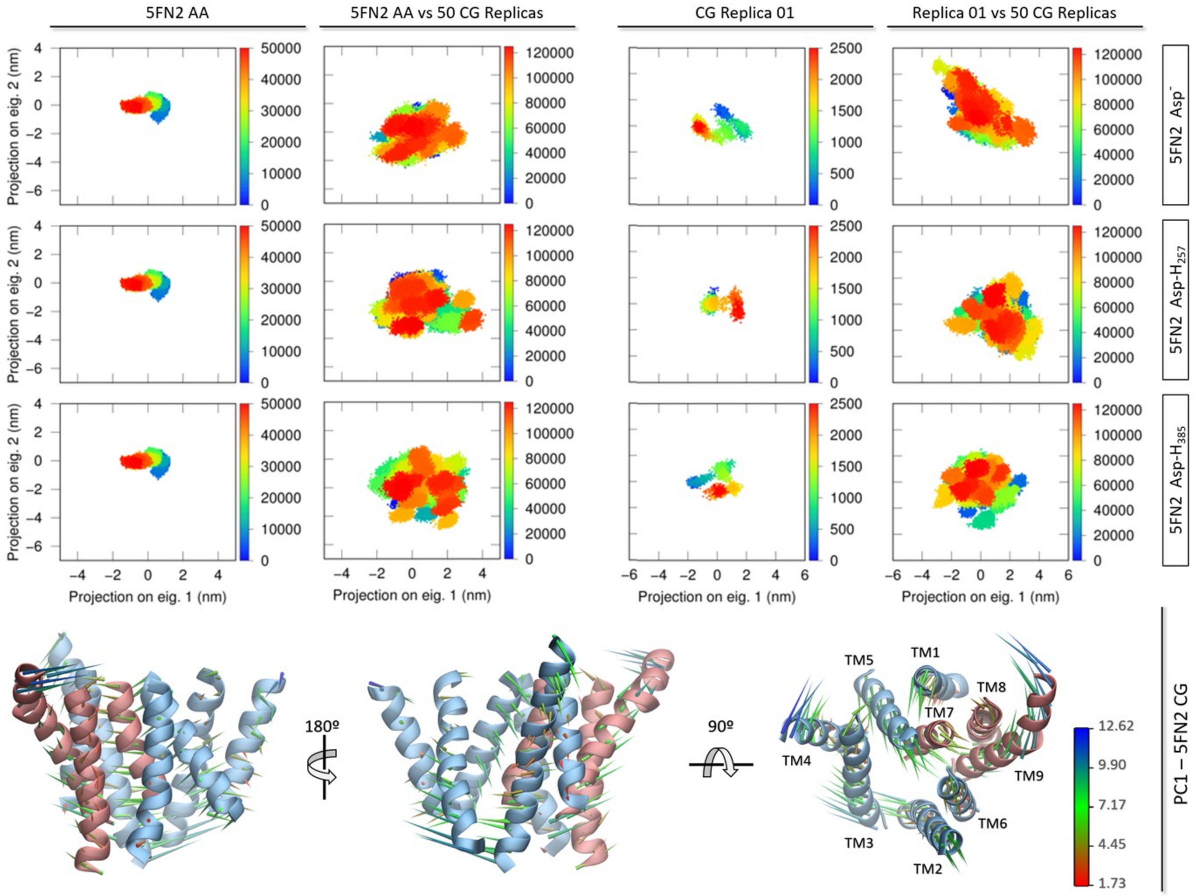


**Fig. S12.** First column, 2D projection of the first and second eigenvectors obtained from the PCA of the last 500 ns of the AA simulated model (5FN2 AA column) for each of the protonated states models: 5FN2 unprotonated, (top), 5FN2 Asp-H257 (middle), and 5FN2 Asp-H385 (bottom). Second column, the fifty CG trajectories projected onto the 5FN2 AA first and second eigenvectors (5FN2 AA vs 50 CG replicas) for the same protonated and unprotonated models. Third column, 2D projection of the first and second eigenvectors obtained from the PCA of one of the Martini CG replicas (CG Replica 01) (out of fifty) for the different protonated and unprotonated models. Fourth column, the projection of the 50 CG trajectories onto the CG Replica 01 first and second eigenvectors (Replica 01 vs 50 CG Replicas) for each of the protonated and unprotonated models. Bottom, PS1 3D-structure models and a porcupine representation of the first eigenvector associated with the transition between *state-1 (inactive)* and *state-2 (active)* conformations of PS1.

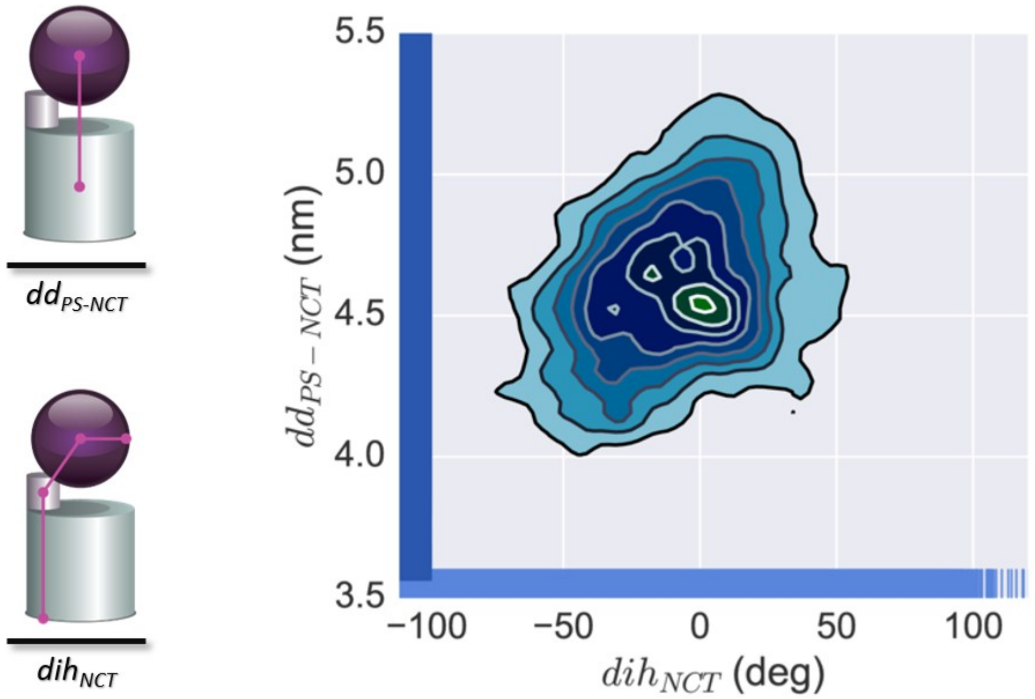


**Fig. S13.** Simulated distribution for 5FN2 and 5FN3 derived CG models in a POPC bilayer projected onto $dd_{PS\text{-}NCT}$ distance and $dih_{NCT}$ rotation.

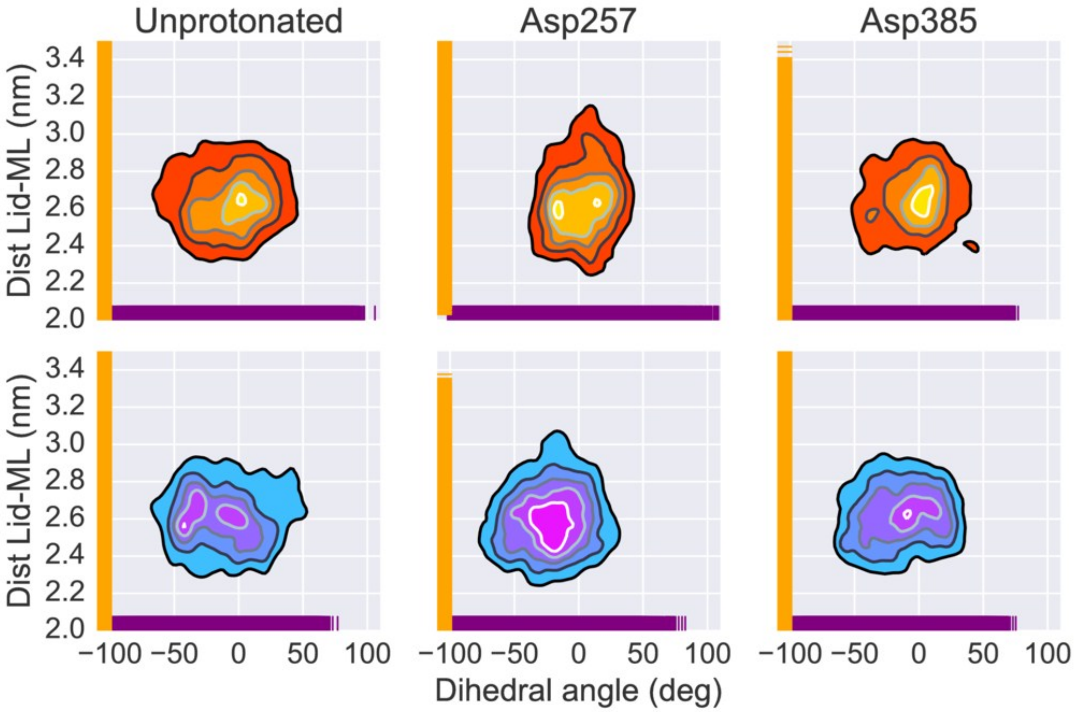


**Fig. S14.** Simulated distributions of 5FN2 (orange) and 5FN3 (blue) derived CG models in a POPC bilayer projected onto (1) the distance between center of geometries of the NCT lid and the NCT major lobe and (2) the NCT rotation (dihedral angle) in the unprotonated and protonated (Asp257 and Asp385) states.

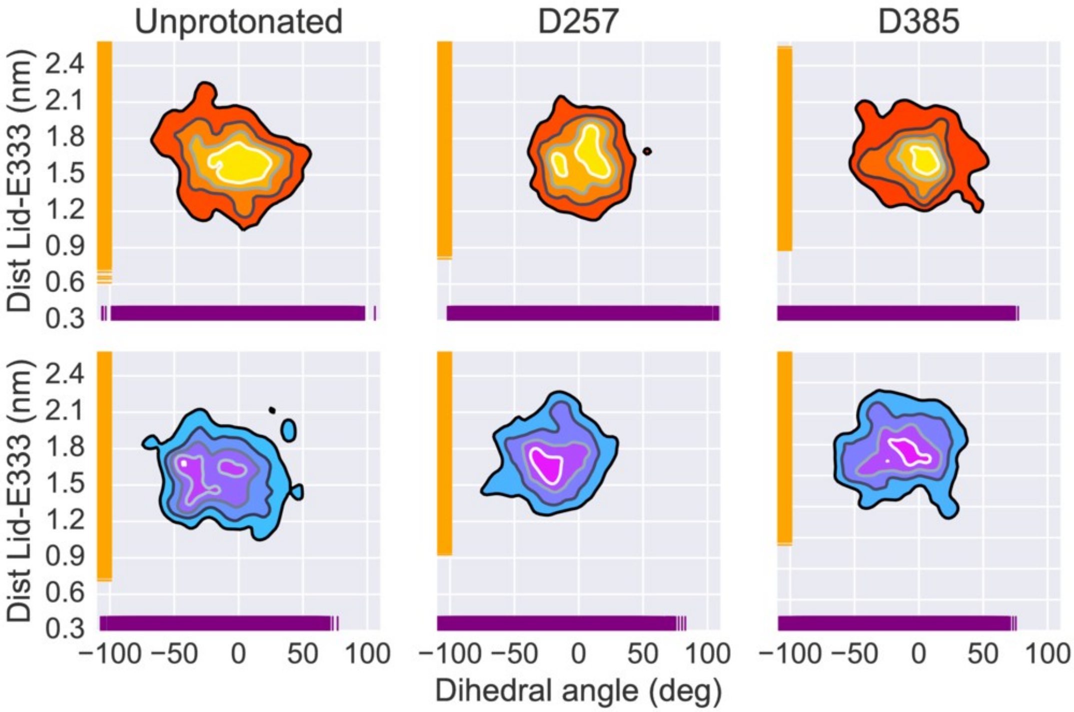


**Fig. S15.** Simulated distributions of 5FN2 (orange) and 5FN3 (blue) derived CG models in a POPC bilayer projected onto (1) the NCT rotation (dihedral angle) and (2) the distance between the NCT lid center of geometry and Glu333, shown for the unprotonated and two protonated (Asp257 and Asp385) states.

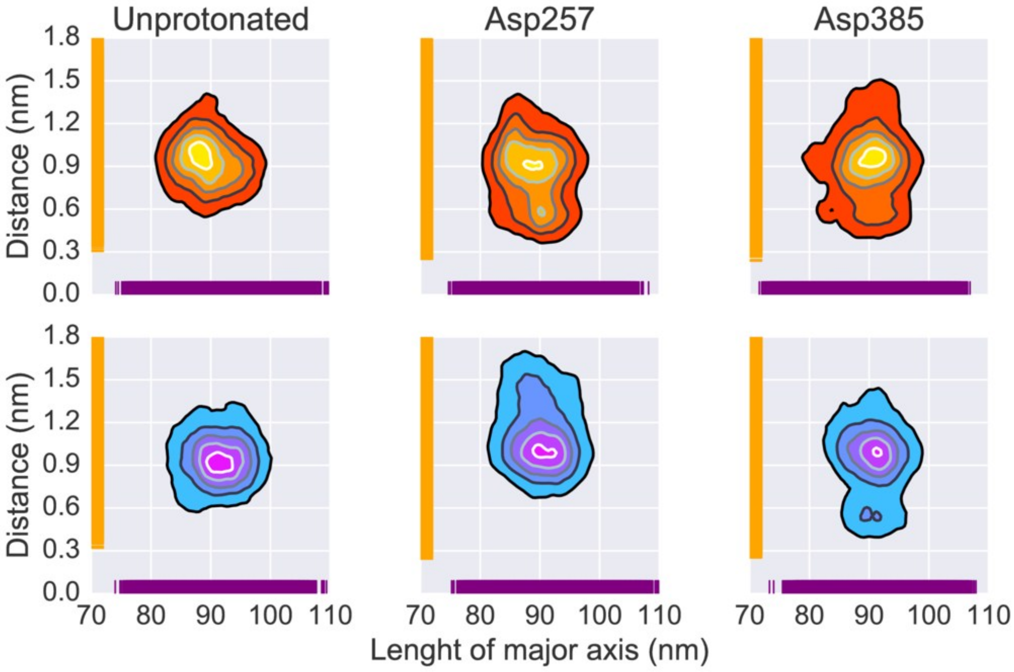


**Fig. S16.** Simulated distributions of 5FN2 (orange) and 5FN3 (blue) derived CG models in POPC bilayer projected onto (1) the length of the major axis of the γ-secretase complex and (2) the distance between the catalytic residues (Asp257 and Asp385) in the unprotonated and protonated (Asp257 and Asp385) states.
